# Green Synthesis of a New Schiff Base Linker and Its Use to Prepare Coordination Polymers

**DOI:** 10.1021/acs.cgd.4c01606

**Published:** 2024-12-25

**Authors:** Maria T. Hayes, Aizhamal Subanbekova, Yassin H. Andaloussi, Alan C. Eaby, Michael J. Zaworotko

**Affiliations:** Department of Chemical Sciences, Bernal Institute, University of Limerick, Limerick V94 T9PX, Republic of Ireland

## Abstract

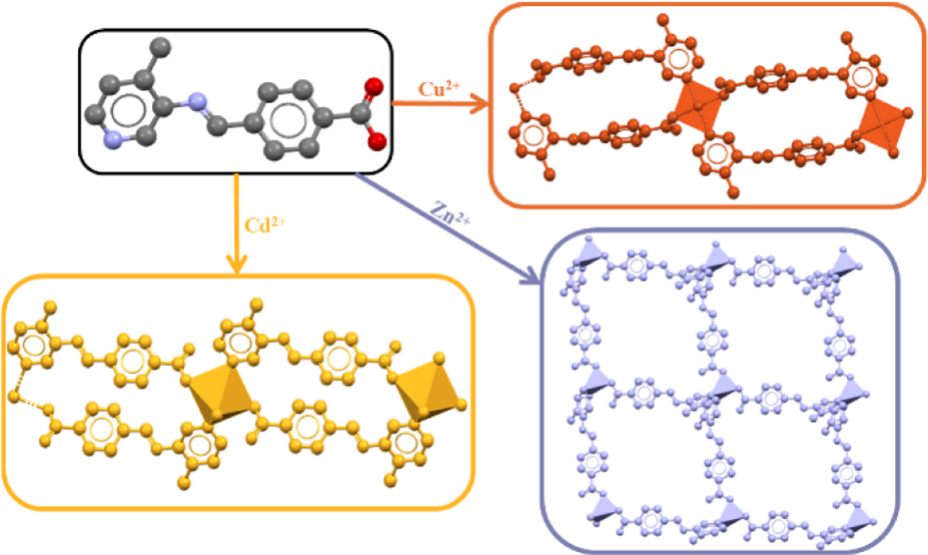

Solid-state synthesis is an approach to organic synthesis that is desirable because it can offer minimal or no solvent waste, high yields, and relatively low energy footprints. Herein, we report the solid-state synthesis of a novel Schiff base, 4-{(*E*)-[(4-methylpyridin-3-yl)imino]methyl}benzoic acid (**4-PIBZ**), synthesized through the reaction of an amine and an aldehyde. **4-PIBZ** was prepared via solvent-drop (water) grinding (SDG) on a multigram scale with 97% yield and was characterized using FTIR, ^1^H NMR, and SCXRD. The pyridyl and carboxylate moiety present in **4-PIBZ** make it suitable for use as a linker ligand and indeed **4-PIBZ** was found to coordinate with Cu(II), Zn(II), and Cd(II) cations, enabling it to serve as a linker ligand for the assembly of coordination polymers. **4-PIBZ** thereby formed 1D (spiro chain) and 2D (square lattice, **sql**, topology) coordination polymers via solvent-induced (layering or slurry) methods. The resulting coordination polymers were characterized though X-ray diffraction (SCXRD, PXRD) and TGA, further demonstrating the utility of green synthesis methods for the preparation of some classes of new linker ligands that can in turn be used for the preparation of coordination polymers.

## Abstract 2

The novel linker ligand {(*E*)-[(4-methylpyridin-3-yl)imino]methyl}benzoic acid (**4-PIBZ**) was synthesized through solid-state synthesis and used to form three coordination polymers, the 2D square lattice coordination network **sql-PIBZ-Zn**, and the 1D coordination polymers **1D-PIBZ-Cu** and **1D-PIBZ-Cd**.

## 1 Introduction

Metal–organic materials (MOMs), including coordination polymers,^1^ metal–organic frameworks (MOFs)^2,3^ are a class of compounds comprised of organic ligands coordinated to metal ions. Owing to the modularity of their components, they offer structural diversity and amenability to design based on crystal engineering principles.^4−6^ Porous MOMs are particularly attractive, as they can act as physisorbents with properties suited for a wide variety of potential applications such as gas storage,^7,8^ gas/vapor separation,^9,10^ atmospheric water harvesting,^11,12^ and drug delivery.^13^ A key aspect of crystal engineering is that once a parent (generation 1) material has been identified, it can enable systematic study of porous MOM platforms (generation 2 materials) that provide insight into structure–function relationships in a manner that cannot yet be easily conducted in any other way.

Although MOMs hold potential for various applications, challenges persist regarding their cost and stability, including their amenability to scale-up.^14,15^ Transitioning from lab-scale to large-scale production is particularly difficult due to the synthetic procedures commonly used in MOM synthesis. These methods typically involve harmful solvents, high temperatures, complex organic ligands, hazardous metal salts, or a combination of these factors.^16^ For example, the use of *N,N*-dimethylformamide (DMF) under solvothermal conditions is frequent due to its tendency to dissolve a broad range of compounds and its ability to form dimethyl amine upon thermal degradation, enabling *in situ* deprotonation of protic ligands such as carboxylic acids.^17^ However, the toxicity of DMF means that it has been restricted in the EU since December 2023, limiting the practicality of MOM manufacture with DMF.^18^ Therefore, the search for alternative nontoxic solvents for sustainable production of MOMs has gained attention in recent years,^19−21^ among which water/aqueous media at room temperature is a preferred option. In addition to solvent and temperature, the synthesis or commercial availability of organic linker ligands and the requisite metal salts can limit the range of scalable MOMs. In terms of organic ligands, options tend to be limited to commercially available linkers or those synthesized via conventional solvent-mediated methods, which typically involve expensive catalysts and wasteful purification processes, further underscoring the need for easily scalable and cost-effective linker synthesis methods.^22^ The selection of metal salts can also pose a challenge in MOM synthesis, as nitrates and chlorides are often favored for their solubility in common solvents, but their hazardous nature raises concerns.^22^

Overall, addressing these challenges requires the development of simpler, more environmentally benign synthetic methods of MOMs through the principles of Green Chemistry.^23,24^ Green synthesis of MOMs has been achieved and in some cases, demonstrated on a large scale. For example, **CALF-20**, **HKUST-1**, and **Al-fumarate** have been synthesized through the use of inexpensive reagents and optimized water/methanol synthetic procedures, successfully overcoming several challenges associated with conventional MOM synthesis.^16,25^ Furthermore, advancements in mechanochemistry have aided large-scale production of MOMs, offering minimal solvent waste, high yields, and scalability,^26,27^ as was demonstrated in the multiton scale production of **Al-fumarate**.^28^ Mechanochemistry has also been increasingly utilized in the synthesis of organic ligands by harnessing mechanical energy to drive reactions,^29,30^ often accelerating these reactions through the addition of trace amounts of solvent, as seen in solvent drop grinding (SDG) or liquid-assisted grinding (LAG).^30,31^ In SDG/LAG, solvents can in effect act as catalysts to enhance yield and efficiency.^32^ An example of a ligand class that is amenable to solid-state synthesis is Schiff bases,^33^ during which a primary amine and an aldehyde undergo condensation to form an imine bond.^34,35^

Linkers can be readily classified into three main categories: N-donor linkers (e.g., 4,4′-bipyridine); O-donor linkers (e.g., terephthalic acid); mixed N/O-donor linkers (e.g., nicotinic acid).^36−39^ Linkers containing both N- and O-donor functionalities,^40,41^ of which nicotinic acid is the parent,^42^ are of interest to crystal engineering as they can form one of the most abundant classes of coordination networks, charge-neutral single-linker networks of general formula ML_2_ (M = divalent metal ion; L = linker).^43^ The ML_2_ composition may result in several topological outcomes as MOMs of diverse structures are known, ranging from 0D complexes to 1D coordination polymers, 2D **sql** (e.g., square lattice, **sql**), and 3D (e.g., diamondoid, **dia**) coordination networks, as well as coordination networks sustained by rod-building blocks (RBBs).^36−38,44−47^ A recent publication of ours addressed the diversity of ML_2_ structures and revealed that **sql**, 1D coordination polymers, **dia** and RBB-based topologies represented 36%, 23%, 17.5%, and 14.5%, respectively, of 1138 reported ML_2_ structures.^43^ Recent publications from our group have demonstrated that such bifunctional linkers can afford materials with high working capacity for methane storage and strong performance for atmospheric water harvesting.^48,49^

**Figure 1:**
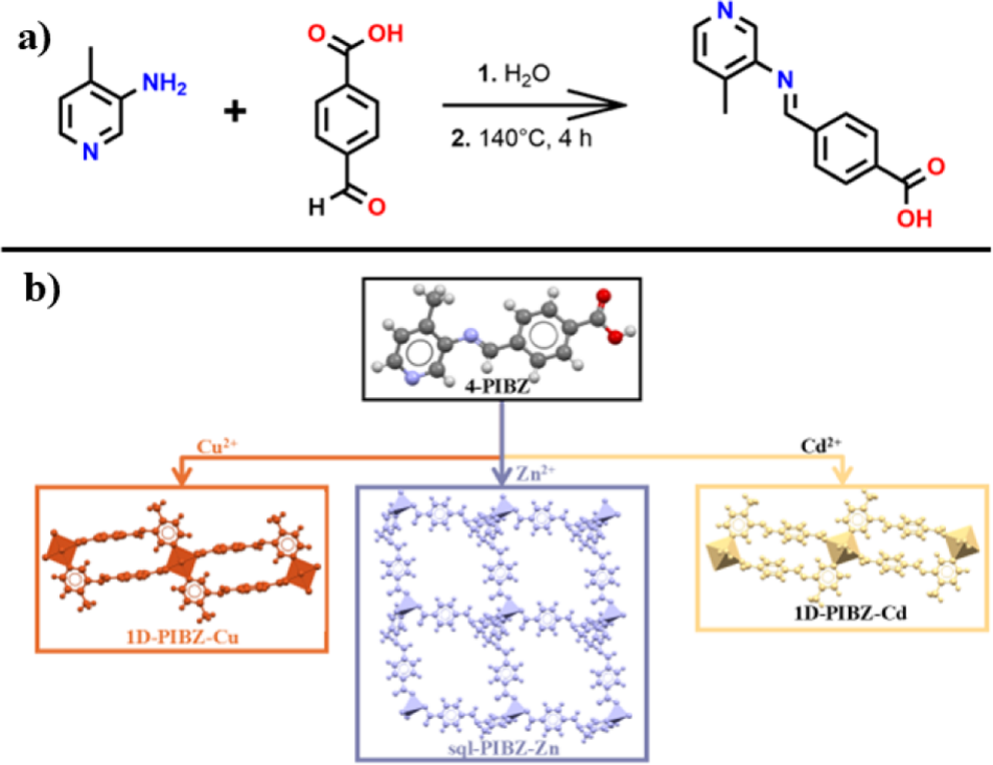
(a) **4-PIBZ** synthesis. (b) Crystal structure of **4-PIBZ** and the three MOMs synthesized from Cu^2+^, Zn^2+^, and Cd^2+^ nitrate and acetate salts.

Herein, we report the details of our study concerning the SDG synthesis of the novel Schiff base linker 4-{(*E*)-[(4-methylpyridin-3-yl)imino]methyl}benzoic acid (**4-PIBZ**) (Figure 1a). **4-PIBZ** was reacted with Zn, Cu and Cd, metal salts to afford **sql-PIBZ-Zn**, **1D-PIBZ-Cu**, and **1D-PIBZ-Cd** (Figure 1b). The structural features of these materials were characterized along with their properties, and the results of our study are detailed herein.

## 2 Experimental Section

### 2.1 Methods and Materials

Reagents and solvents were purchased from Sigma-Aldrich, Thermo Scientific, Fluorochem, or TCI and used without further purification. Thermogravimetric analysis (TGA) was performed on a TA Instruments Q50 TG with a heating rate of 10 °C min^–1^ from room temperature up to 500 °C. Fourier Transform infrared (FTIR) spectra were collected on a PerkinElmer Spectrum 100 spectrometer with a Universal ATR accessory from 650 to 4000 cm^–1^. Powder X-ray diffraction (PXRD) data was measured with a PANanalytical Empyrean diffractometer (40 kV, 40 mA, Cu K_α_,1,2, λ = 1.5418 Å) in Bragg–Brentano geometry. Crystal structures were determined by single crystal X-ray diffraction (SCXRD) data collected at 150 K with either Cu K_α_ (λ = 1.5418 Å) (for **4-PIBZ**, **sql-PIBZ-Zn**, and **1d-PIBZ-Cd**) or Mo radiation (λ = 0.71073 Å) (for **1D-PIBZ-Cu**). The Bruker D8 Quest fixed-chi diffractometers used were equipped with Bruker APEX-II CCD detectors and a nitrogen-flow Oxford Cryosystem attachment. Gas sorption studies were performed using a Micromeritics Tristar II 3030 instrument. Water vapor sorption isotherm determination was performed using an Adventure Dynamic Vapor Sorption (DVS) instrument manufactured by Surface Measurement Systems. Upscaled synthesis of **4-PIBZ** was performed in a Retsch MM400 ball mill using two 10 mm stainless steel balls at 25 Hz. Full experimental details are presented in Supporting Information.

### 2.2 4-PIBZ Solid State Synthesis

The two starting materials (3-amino-4-methylpyridine and 4-formylbenzoic acid) along with catalytic amounts of H_2_O were ground using a mortar and pestle for milligram-scale synthesis and in a ball mill for multigram-scale synthesis. Heating the resulting powder to 140 °C yielded a pale-yellow powder (97% yield). M.P. 194 °C; FTIR 1627 cm^–1^ (*v*_CH=N_) (Figure S1). ^1^H NMR (400 MHz, DMSO-*d*_6_) δ 2.31 (s, 3H), 7.29 (d, 1H), 8.08 (m, 4H), 8.25 (s, 1H), 8.32 (d, 1H), 8.66 (s, 1H) (Figure S2).

### 2.3 Single Crystal Preparation

Single crystals of **sql-PIBZ-Zn**, **1D-PIBZ-Cu**, and **1D-PIBZ-Cd** were obtained at room temperature by layering **4-PIBZ** with Zn(NO_3_)_2_·6H_2_O, Cu(NO_3_)_2_·3H_2_O, or Cd(NO_3_)_2_·4H_2_O. **4-PIBZ** was dissolved in MeOH while the metal salts were dissolved in water. Solutions were layered in the following order, metal nitrate in H_2_O, MeOH/H_2_O buffer and **4-PIBZ** in MeOH. Single crystals of **4-PIBZ** were prepared by recrystallization from MeOH (slow evaporation) of the powdered reaction product sample obtained from ball-milling after heating.

### 2.4 Scale-Up Synthesis of sql-PIBZ-Zn and 1D-PIBZ-Cu

Bulk syntheses were conducted via slurry in a MeOH/H_2_O solution under ambient conditions. Two metal sources were used for Zn^2+^ and Cu^2+^, the nitrate or acetate salts.

## 3 Results and Discussion

### 3.1 4-PIBZ

**Figure 2:**
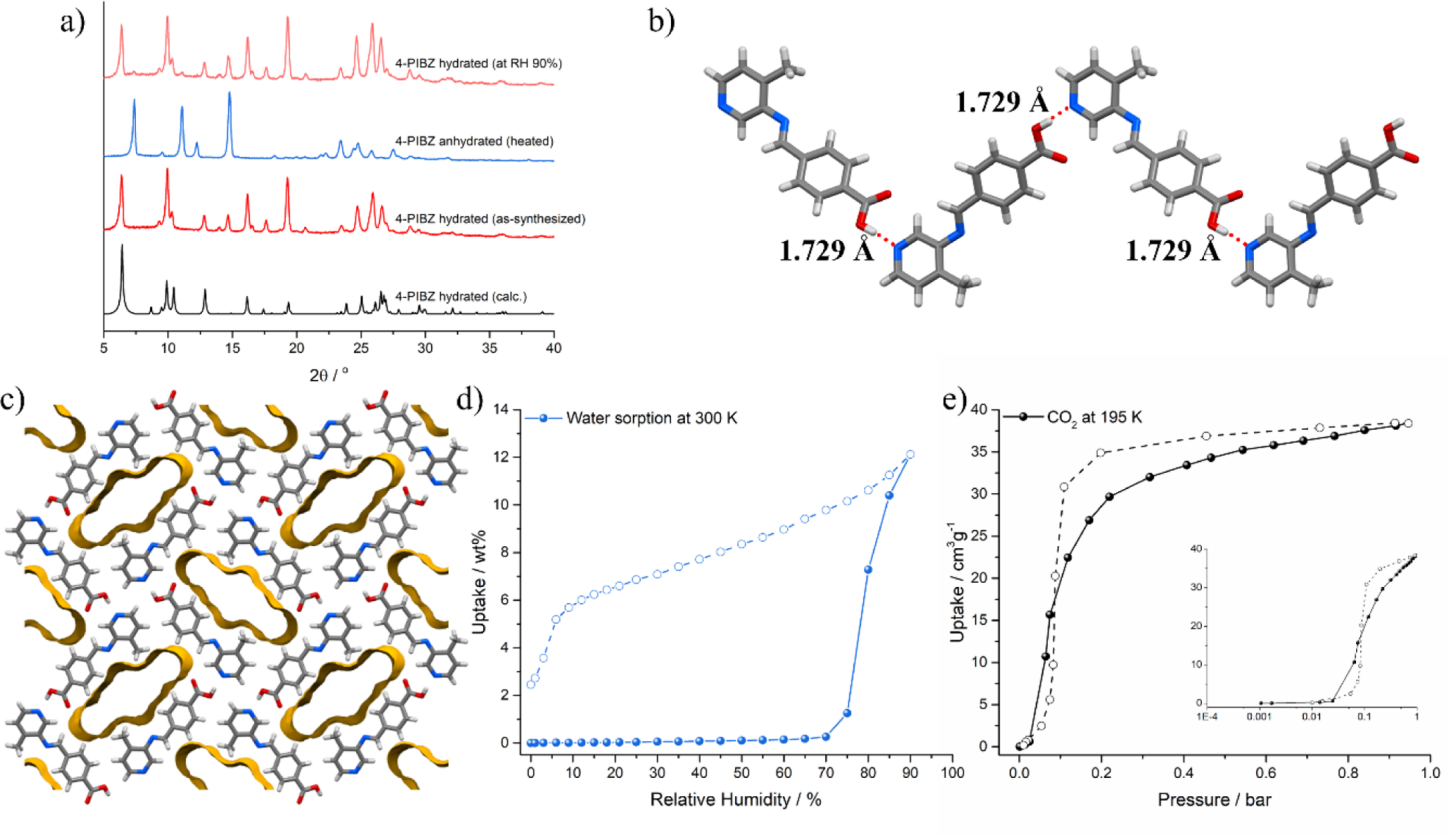
(a) PXRD patterns of the hydrate and anhydrate phases of **4-PIBZ**; (b) 1D zigzag hydrogen bonded chain structure of **4-PIBZ**; (c) rectangular channels of solvated (MeOH and/or H_2_O) **4-PIBZ** revealing pores along the *a*-axis. (d) DVS analysis on **4-PIBZ** collected at 300 K, and (e) CO_2_ sorption analysis on **4-PIBZ** at 195 K.

The linker ligand **4-PIBZ**, which was previously unreported according to searches conducted using SciFinder and ConQuest (CSD version 2024.2.0)^50^ in July 2024, was synthesized by solvent drop grinding (SDG) at room temperature (Figures S1–S3). Thermogravimetric analysis (TGA) of the as-synthesized **4-PIBZ** powder revealed a mass loss of 9.45% at 140 °C equivalent to *ca*. 1.4 molecules of water per formula unit (Figure S4), indicating that the as-synthesized phase could be a hydrate. Powder X-ray diffraction (PXRD) was conducted before and after heating to 140 °C, as well as differential scanning calorimetry (DSC) (Figure S5) was consistent with a phase transformation between hydrate and anhydrate forms of **4-PIBZ** (Figure 2a). Soaking in water or exposure to high humidity caused reversion to the hydrate phase, as evidenced by PXRD (Figure 2a). To further investigate these phase transformations of **4-PIBZ**, single crystals were prepared by the slow evaporation of **4-PIBZ** from MeOH, yielding parallel piped crystals that exhibit channels containing disordered MeOH or H_2_O molecules following crystallization in the monoclinic space group *P*2_1_/n (Table S1). **4-PIBZ** was observed to be present in a nonplanar configuration with an angle of 34.85° subtended between the pyridyl and phenyl rings. Specifically, the dihedral angles between the imine bond and centroid of the phenyl or pyridinyl ring are 122.61° and 120.14°, respectively (Figure S7). The imine bond length of 1.266 Å and torsion angle of 179.72° are, according to our CSD search, common for imines (Figure S8). Hydrogen bonding between adjacent **4-PIBZ** carboxylic acid and pyridyl groups is sustained by the expected^51^ R_2_^2^(8) supramolecular heterosynthon^52,53^ with an O···N distance of 2.567 (3) Å, affording a zigzag chain motif (Figure 2b).^54^ Additional short contacts between pyridyl CH hydrogen atoms and carboxylate oxygen groups sustain channels along the *a*-axis with a void space of 24.2% of the unit cell volume (calculated with a probe radius of 1.2 Å, Figure 2c). Although the solvent molecules were not crystallographically resolvable, the implementation of SQUEEZE by the OLEX2 implementation indicated an electron density consistent with *ca*. 1.5 MeOH molecules per formula unit. This is also consistent with the *ca*. 1.4 molecules of H_2_O indicated by TGA analysis of water SDG-synthesized **4-PIBZ** (Figure S4). DSC data of anhydrous **4-PIBZ** revealed an endothermic peak at 192 °C, likely corresponding to thermal decomposition, as also suggested by TGA analysis (Figures S4 and S9). The calculated PXRD pattern from the single crystal structure of solvated **4-PIBZ** is a good match with the experimental PXRD pattern from a sample obtained from water SDG. Attempts to obtain SCXRD data of the anhydrate form of **4-PIBZ** were unsuccessful as crystals of hydrated **4-PIBZ** fractured into a microcrystalline powder upon solvent removal. Variable-temperature PXRD (VT PXRD) analysis of the anhydrate form of **4-PIBZ** indicated structural stability up to 160 °C (Figure S10) whereas the hydrated form gradually transformed into the anhydrate phase starting at 40 °C, being complete by 120 °C (Figure S11).

The propensity for **4-PIBZ** to transform between the hydrate/solvate and anhydrate phases prompted further investigation of this behavior. Dynamic water vapor sorption (DVS) data was collected after activating a sample of **4-PIBZ** by heating under dry air flow at 373 K. The water sorption isotherm collected at 300 K exhibited a switching type F–IV isotherm,^48^ with a step at 75% relative humidity (RH) and saturation uptake of 12.12 wt % corresponding to 1.62 equiv of water (Figure 2d). The desorption profile displayed large hysteresis, with a steep decline in uptake at ∼3% RH. Full desorption was not achieved, leaving 2.45 wt % (*ca*. 0.3 equiv) of water present in the structure at 0% RH. These results are consistent with thermal analysis experiments, which indicated that heating to 140 °C is necessary to remove all water molecules of hydration from the hydrate phase of **4-PIBZ** (Figure S4). A similar switching phenomenon was observed for the 195 K CO_2_ sorption isotherm of **4-PIBZ** after activation at 100 °C under a vacuum, revealing a sharp step indicative of switching behavior between open-pore and closed-pore phases (Figure 2e). The resulting single-step type F–IV isotherm displayed a CO_2_ induced gate-opening event at 0.02 bar and saturation uptake of 38.4 cm^3^g^–1^ (1.7 mmol g^–1^). The desorption profile showed a slight increase in uptake, likely due to gradual warming of the dry ice–acetone bath during the measurement.

### 3.2 sql-PIBZ-Zn

**Figure 3:**
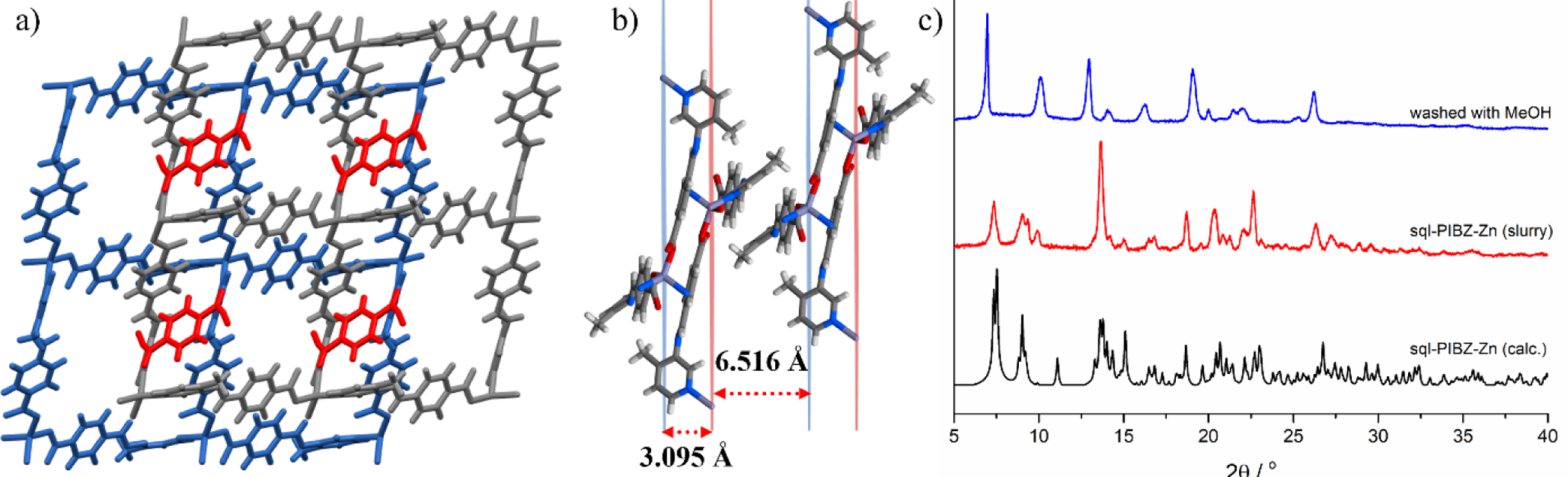
(a) Structure of **sql-PIBZ-Zn** viewed along the *a*-axis. Symmetrically independent **sql** nets are labeled in blue and gray, and the 4-formylbenzoic acid guest is in red. (b) **sql-PIBZ-Zn** viewed along the *c*-axis revealing interlayer spacing between adjacent coordination network pairs. (c) PXRD patterns of **sql-PIBZ-Zn** before and after washing with MeOH.

Layering a MeOH/H_2_O solution of NaOH-deprotonated **4-PIBZ** and zinc nitrate (2:1) afforded crystals of **sql-PIBZ-Zn** suitable for SCXRD analysis (Figure 3a). Bulk samples of **sql-PIBZ-Zn** were synthesized by slurrying **4-PIBZ** with zinc nitrate or the more environmentally benign zinc acetate, with PXRD patterns, FTIR spectra, and TGA thermograms indicating that the same microcrystalline product had been produced (Figures S12–S14). SCXRD revealed that **sql-PIBZ-Zn** had crystallized in the triclinic *P*1 space group with a Zn^2+^ cation and two independent **4-PIBZ** anions in the asymmetric unit, affording the expected ML_2_ composition. Zn^2+^ tetrahedrally coordinates to two pyridyl and two carboxylate groups, which, combined with the nonlinear shape of the **4-PIBZ** linker, resulted in a 2D square lattice, **sql**, topology coordination network (Figure 3a). In addition to the coordination network, 4-formylbenzoic acid was found to be present as a guest, along with one crystallographically resolvable water molecule. Additional solvent molecules that could not be resolved crystallographically were handled through the OLEX2 implementation of SQUEEZE, indicating electron density consistent with the presence of *ca*. 10 additional H_2_O molecules per unit cell. The presence of 4-formylbenzoic acid could have been a residue from the SDG synthesis, thereby explaining the unexpectedly high temperature of solvent loss in the as-synthesized samples of **4-PIBZ**, or from zinc-catalyzed hydrolysis of **4-PIBZ** prior to MOM formation. TGA of **sql-PIBZ-Zn** revealed a mass loss of 19.5 wt % at 235 °C consistent with removal of 4-formylbenzoic acid (Figure S14). 4-Formylbenzoic acid was also removed by soaking crystals of **sql-PIBZ-Zn** in MeOH overnight (Figure 3c). TGA analysis after soaking in MeOH revealed a mass loss of MeOH (10.4 wt% at 65 °C) followed by decomposition of the material (Figure S15). In the absence of guest molecules, the structure was calculated to have 36.9% void space because of continuous 3D channels along the [100], [010], [110], and [101] directions (Figure S16). The 2D **sql** sheets formed non-interpenetrating interlocking pairs with Zn–Zn layer distances of 3.0953 (19) Å (within pairs) and 6.516(2) Å (between pairs) (Figure3b). Replacement of zinc nitrate with zinc acetate also yielded **sql-PIBZ-Zn** according to PXRD and TGA data (Figures S12 and S14). To assess the porosity of **sql-PIBZ-Zn**, low-pressure CO_2_ adsorption measurements were conducted at 195 K, revealing an uptake of 10.6 cm^3^ g^–1^ (Figure S17).

### 3.3 1D-PIBZ-Cu

**Figure 4:**
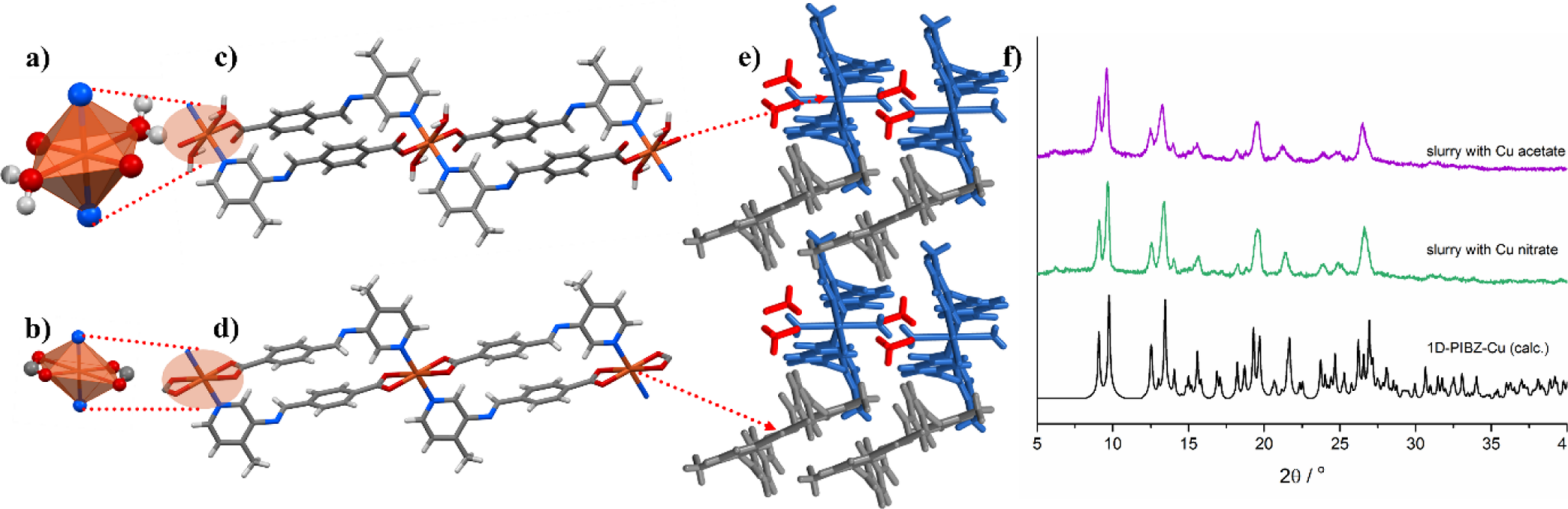
Cu^2+^ environments in **1D-PIBZ-Cu**: (a) distorted octahedral Cu1 and (b) pseudo square planar Cu2. **1D-PIBZ-Cu** spiro loop chains with (c) octahedral Cu1 and two coordinated aqua ligands and (d) square planar Cu2. (e) Packing of the aqua-coordinated (blue) and ML_2_ (gray) chains with the MeOH guest molecules (red) as viewed along the *b*-axis. (f) PXRD patterns of the product of a **1D-PIBZ-Cu** slurry.

A 1D coordination polymer with spiro loops, **1D-PIBZ-Cu**, was obtained by the reaction of copper nitrate and **4-PIBZ** (Figure 4a). **1D-PIBZ-Cu** crystallized in the triclinic space group *P*1̅ and is a cocrystal comprised of two 1D coordination polymers with different compositions, each asymmetric unit comprising half of each 1D polymer (*Z*′ = 0.5) (Figures S18–S19). The geometry of the Cu^2+^ cations in **1D-PIBZ-Cu** revealed axial elongations due to the Jahn–Teller effect, with Cu–O (carboxylate) and Cu–OH_2_ distances of 2.8438(15) and 2.6083(16) Å for Cu1 and Cu2, respectively (Figures S20–S21). In effect, Cu1 exhibits pseudo square planar geometry, whereas Cu2 adopts distorted octahedral geometry. One of the coordination polymers is comprised of 4-connected square planar copper centers coordinated to two pyridyl and two carboxylate groups to form a 1D structure of formula [ML_2_]_*n*_ (Figure 4b). The second 1D coordination polymer is composed of an octahedrally coordinated copper center, which, in addition to two pyridyl and two carboxylate groups, coordinates to two aqua ligands (Figure 4a) of formula [ML_2_(H_2_O)_2_]_*n*_. The aqua ligands form hydrogen bonds to carboxylate carbonyl groups of adjacent aqua-coordinated 1D polymers (Figure 4c,d) through an R_4_^4^(8) hydrogen bonding pattern with aqua oxygen···carboxyl oxygen (O4···O5) distances of 2.842(2) Å. Further hydrogen bonding, with an O5···O6 distance of 2.755(3) Å, occurs between the coordinated aqua ligand and a methanol molecule with partial chemical occupancy freely refined to 0.696. The two compositionally different coordination polymers pack into an alternating bilayer structure that stack along the *b*-axis (Figure 4e). The occurrence of two chemically independent copper-based coordination polymers involving octahedral and square-planar geometries has previously been reported by Batten et al., who reported a combination of a 2D network based on octahedral copper and a 1D polymer based on square-planar copper occurring in the same structure.^55^ Structures specifically involving both (μ_2_-η^1^:η^1^)-carboxylate connected and (μ_1_-η^1^)-carboxylate connected copper ions were identified through a CSD search (Figure S22) that yielded only two hits: refcodes GAQYUT and JEQZUE. JEQZUE features two distinct Cu^2+^ sites (square planar and octahedral) coordinated by a bifunctional linker, 4-(1*H*-imidazole-4-yl) benzoate, forming a 2D **hcb** network (Figure S23).^56^ GAQYUT is comprised of 1,3-bis(4-pyridyl)propane linkers that coordinate to three different Cu^2+^ nodes (square pyramidal, square planar, and octahedral), creating a 1D chain, which self-assembles into a triple-stranded braid via H-bonding (Figure S24).^57^

An upscaled synthesis was performed by slurrying at RT in MeOH/H_2_O using either copper nitrate or copper acetate, with both slurries yielding powders with PXRD patterns that match that of **1D-PIBZ-Cu** (Figure 4f). TGA revealed **1D-PIBZ-Cu** to be thermally stable up to *ca*. 260 °C (Figure S25). To evaluate porosity, a low-pressure CO_2_ adsorption measurement was performed at 195 K, showing an uptake of 7.3 cm^3^ g^–1^ at a saturation pressure (Figure S26).

### 3.4 1D-PIBZ-Cd

**Figure 5:**
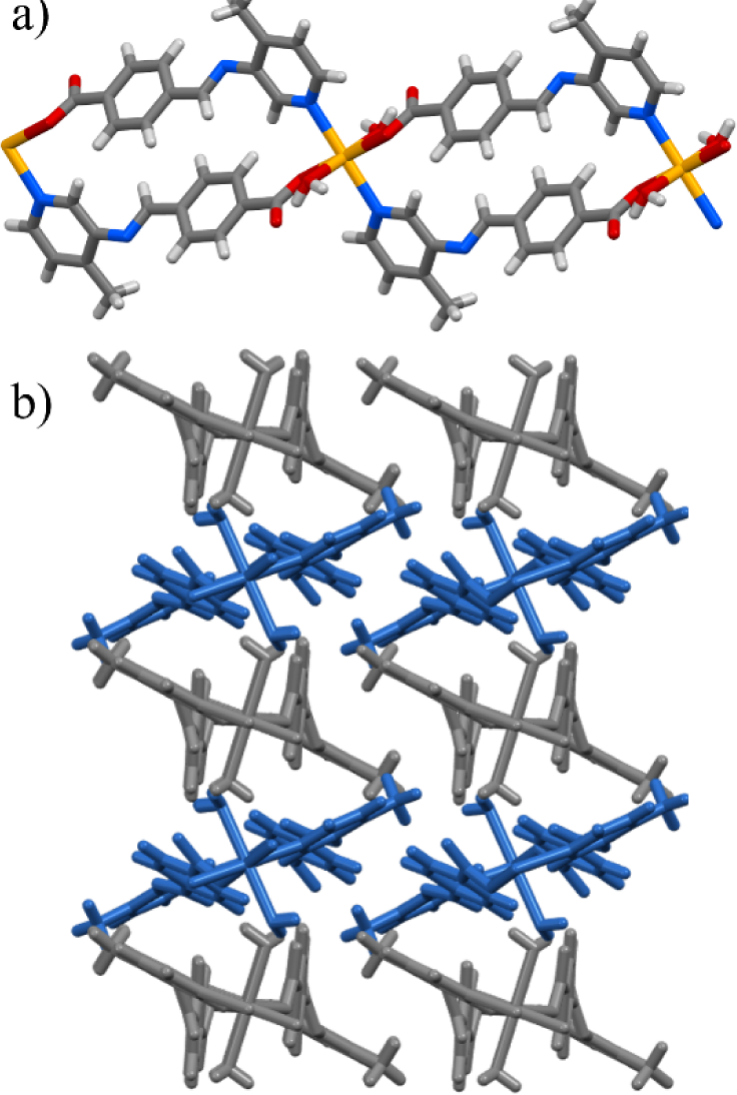
(a) Structure of 1D spiral loop containing Cd^2+^, two aqua ligands, and two molecules of **4-PIBZ** as the organic linker. (b) **1D-PIBZ-Cd** polymer packing as viewed along the [101] direction.

**1D-PIBZ-Cd** forms a similar structure to that of **1D-PIBZ-Cu**, wherein two crystallographically independent 1D coordination polymers cocrystallize in the triclinic space group *P*1̅, forming spiro loops with each asymmetric unit possessing half of each 1D polymer (Figure 5a). In contrast to **1D-PIBZ-Cu**, both 1D polymer chains have the same ML_2_(H_2_O)_2_ composition (Figure S27). Each independent 1D coordination polymer alternates hydrogen bonding between each symmetrically independent polymer along the *b*-axis (Figure 5b). The lack of symmetry between each 1D polymer arises from the aqua ligands bound to Cd1 acting exclusively as a hydrogen bond donor to Cd2 aqua ligands (O3···O6) and Cd1 carbonyl oxygens (O3···O2) with distances of 2.774(4) and 2.689(4) Å, respectively. The aqua ligands bound to Cd2 function as hydrogen bond donors to the Cd2-coordinated carboxylate oxygens (O2···O1) and Cd1-coordinated carboxylate oxygens (O6···O1) with distances of 2.628(3) Å and 2.759(3) Å, respectively, leaving the remaining Cd2-coordinated carboxylate oxygen (O4) without further hydrogen bonding.

From a crystal engineering perspective, we had anticipated that the use of a mixed N/O-donor linker would produce MOMs with an ML_2_ composition. In this case, two of the most commonly encountered topologies were observed: a 2D **sql** coordination network and two 1D spiro loop structures.^43^ While the synthesis of these ML_2_ structures was anticipated for **4-PIBZ**, their gas sorption properties did not align with the amount of available guest-accessible space in their solvated structures. For example, **sql-PIBZ-Zn** has a guest-accessible space of 36.9% (633.90 Å^3^, using a probe radius of 1.2 Å) but only achieves a CO_2_ uptake of 10.6 cm^3^ g^–1^ at saturation pressure, which is consistent with surface sorption. Similarly, **1D-PIBZ-Cu** displayed CO_2_ uptake of 7.3 cm^3^ g^–1^ at saturation pressure that does not match the amount of methanol present in the structure (6.1% or 78.94 Å^3^ guest-accessible space). This is perhaps surprising since other coordination networks having similar structural features (1D spiro loops or 2D **sql** nets) have been shown to exhibit interesting gas sorption properties. For instance, 3D CNs involving bent N-donor linkers in spiro loop structures exhibited guest-induced transformations and strong gas separation properties.^58−60^ In addition, 2D CNs based mixed N/O-donor linkers showed gas sorption coinciding with structural transformations, which were driven by linker motion (twisting, bending, rotation).^47^

Careful analysis of the crystal structures was performed in order to gain insight into the unexpected sorption behavior in comparison to that in related structures from the literature. The 2D **sql-PIBZ-Zn** structure, although theoretically capable of interlayer expansion in response to guest molecules or linker motion, did not undergo a CO_2_ induced phase change at 195 K. We attribute this to being unable to overcome interlayer interactions, *i.e*., 3.78 Å from π–π stacking interactions and 3.34 Å (O4···C1) from H-bonded short contacts (Figure S28). In comparison to other 2D CNs, these interactions are relatively strong, which could indicate that CO_2_ guest molecules are unable to compensate for the loss of these interactions during a phase transformation (Table S2). Regarding **1D-PIBZ-Cu**, even though the structure contains spiro loops, the methanol guest molecules in the as-synthesized phase are located outside these loops (Figure 4e), indicating that the loop itself is too small to accommodate guest molecules. Indeed, the loops exhibit maximum distances of 5.17 and 5.32 Å between opposite linkers (π–π stacking), which makes spiro loops of **1D-PIBZ-Cu** too small to fit a guest molecule (Table S2, Figure S29). Additionally, 3.67 Å contacts from π–π stacking interactions between individual chains, along with hydrogen bonding between aqua ligands and carboxylate moieties at (O6···O7 = 2.848 Å, Figures S30–S32), contribute to the closed packing and rigid nature of the structure. These numbers can be compared to reported CNs such as WOBCOH,^61^ in which distances of 8.825 Å between aromatic rings in its loop structure enabled adsorption of 35 cm^3^ g^–1^ of CO_2_ at 275 K. Other linkers such as 4,4′-dipyridylethane^59^ and 4,4′-dipyridylpropane^60^ have enabled the generation of spiro loops with intrinsic porosity and exceptional sorption performance for sorbates such as acetylene and propene, respectively.

## 4 Conclusions

In this work, we report the novel linker ligand **4-PIBZ**, which was synthesized via a mechanochemical method that afforded a 97% yield in an easily scalable process. Three coordination polymers were synthesized using **4-PIBZ** as a linker ligand with Zn, Cu, and Cd metal cations to form 2D (**sql-PIBZ-Zn**) and 1D (**1D-PIBZ-Cu**, **1D-PIBZ-Cd**) structures. These coordination polymers were obtained through RT layering/slurry methods in MeOH/H_2_O and were characterized by XRD. **sql-PIBZ-Zn** was found to form a 2D **sql** topology coordination network with an unexpected guest, 4-formylbenzoic acid, while **1D-PIBZ-Cu** and **1D-PIBZ-Cd** formed 1D spiral loop coordination polymers, both with two symmetrically independent polymer chains. Slurrying with metal nitrates or metal acetates also produced **sql-PIBZ-Zn** and **1D-PIBZ-Cu**. The results reported herein highlight the use of crystal engineering strategies for the design and synthesis of new families of organic linkers and coordination networks and align materials discovery with the principles of green chemistry.

## Supporting Information Available

Experimental details of synthesis and characterization, supplementary characterization results, additional figures, tables and data analysis (PDF)

## Abbreviations

**4-PIBZ**: 4 {(*E*)-[(4-methylpyridin-3-yl)imino]methyl}benzoic acid
CN: coordination network
DMF: *N,N*-dimethylformamide
FTIR: Fourier transform infrared spectra
DVS: dynamic vapor sorption
SDG: solvent drop grinding
**sql**: square lattice
**dia**: diamondoid
MOF: metal–organic framework
MOM: metal–organic material
VTPXTD: variable temperature powder X-ray diffraction
PXRD: powder X-ray diffraction
SCXRD: single-crystal X-ray diffraction
TGA: thermogravimetric analysis
RBB: rod-building blocks

## References

[ref1] Kitagawa S.; Kitaura R.; Noro S.-I. Functional Porous Coordination Polymers. Angew. Chem., Int. Ed. 2004, 43 (23), 2334–2375. 10.1002/anie.200300610. 15114565

[ref2] Zhou H.-C. J.; Kitagawa S. Metal–Organic Frameworks (MOFs). Chem. Soc. Rev. 2014, 43 (16), 5415–5418. 10.1039/C4CS90059F. 25011480

[ref3] Furukawa H.; Cordova K. E.; O’Keeffe M.; Yaghi O. M. The chemistry and applications of metal-organic frameworks. Science 2013, 341 (6149), 1230444 10.1126/science.1230444. 23990564

[ref4] Desiraju G. R. Crystal engineering: The design of organic solids. Mater. Sci. Monogr., 1989, 54.

[ref5] Bhattacharya S.; Peraka K. S.; Zaworotko M. J. The role of hydrogen bonding in co-crystals Co-Crystals: Preparation, Characterization And Applications Royal Society of chemistry 2018 24 33 10.1039/9781788012874-00033

[ref6] Perry J. J. IV; Perman J. A.; Zaworotko M. J. Design and synthesis of metal–organic frameworks using metal–organic polyhedra as supermolecular building blocks. Chem. Soc. Rev. 2009, 38 (5), 1400–1417. 10.1039/B807086P. 19384444

[ref7] Wang W.; Chen D.; Li F.; Xiao X.; Xu Q. Metal-organic-framework-based materials as platforms for energy applications. Chem 2024, 10 (1), 86–133. 10.1016/j.chempr.2023.09.009.

[ref8] Xiao C.; Tian J.; Chen Q.; Hong M. Water-stable metal–organic frameworks (MOFs): Rational construction and carbon dioxide capture. Chem. Sci. 2024, 15 (5), 1570–1610. 10.1039/D3SC06076D. 38303941 PMC10829030

[ref9] Czaja A. U.; Trukhan N.; Müller U. Industrial applications of metal–organic frameworks. Chem. Soc. Rev. 2009, 38 (5), 1284–1293. 10.1039/b804680h. 19384438

[ref10] Zhang X.; Li Y.; Li J.-R. Metal-organic frameworks for multicomponent gas separation. Trends Chem. 2024, 6 (1), 22–36. 10.1016/j.trechm.2023.11.001. 39183183

[ref11] Hanikel N.; Prévot M. S.; Fathieh F.; Kapustin E. A.; Lyu H.; Wang H.; Diercks N. J.; Glover T. G.; Yaghi O. M. Rapid Cycling and Exceptional Yield in a Metal-Organic Framework Water Harvester. ACS Cent. Sci. 2019, 5 (10), 1699–1706. 10.1021/acscentsci.9b00745. 31660438 PMC6813556

[ref12] Shi L.; Kirlikovali K. O.; Chen Z.; Farha O. K. Metal-organic frameworks for water vapor adsorption. Chem 2024, 10 (2), 484–503. 10.1016/j.chempr.2023.09.005.

[ref13] Pettinari C.; Marchetti F.; Mosca N.; Tosi G.; Drozdov A. Application of metal – organic frameworks. Polym. Int. 2017, 66 (6), 731–744. 10.1002/pi.5315.

[ref14] Kumar S.; Jain S.; Nehra M.; Dilbaghi N.; Marrazza G.; Kim K.-H. Green synthesis of metal–organic frameworks: A state-of-the-art review of potential environmental and medical applications. Coord. Chem. Rev. 2020, 420, 213407 10.1016/j.ccr.2020.213407.

[ref15] Ryu U.; Jee S.; Rao P. C.; Shin J.; Ko C.; Yoon M.; Park K. S.; Choi K. M. Recent advances in process engineering and upcoming applications of metal–organic frameworks. Coord. Chem. Rev. 2021, 426, 213544 10.1016/j.ccr.2020.213544. 32981945 PMC7500364

[ref16] Chakraborty D.; Yurdusen A.; Mouchaham G.; Nouar F.; Serre C. Large-Scale Production of Metal–Organic Frameworks. Adv. Funct. Mater. 2024, 34 (n/a), 2309089 10.1002/adfm.202309089.

[ref17] Muzart J. N,N-Dimethylformamide: Much more than a solvent. Tetrahedron 2009, 65 (40), 8313–8323. 10.1016/j.tet.2009.06.091.

[ref18] Sherwood J.; Albericio F.; de la Torre B. G. *N,N*-Dimethyl Formamide European Restriction Demands Solvent Substitution in Research and Development. ChemSusChem 2024, 17 (8), e202301639 10.1002/cssc.202301639. 38200662

[ref19] Morelli Venturi D.; Campana F.; Marmottini F.; Costantino F.; Vaccaro L. Extensive Screening of Green Solvents for Safe and Sustainable UiO-66 Synthesis. ACS Sustainable Chem. Eng. 2020, 8 (46), 17154–17164. 10.1021/acssuschemeng.0c05587.

[ref20] Fu J.; Wu Y.-N. A Showcase of Green Chemistry: Sustainable Synthetic Approach of Zirconium-Based MOF Materials. Chem. - Eur. J. 2021, 27 (39), 9967–9987. 10.1002/chem.202005151. 33955075

[ref21] Chen J.; Shen K.; Li Y. Greening the Processes of Metal–Organic Framework Synthesis and their Use in Sustainable Catalysis. ChemSusChem 2017, 10 (16), 3165–3187. 10.1002/cssc.201700748. 28589626

[ref22] Julien P. A.; Mottillo C.; Friščić T. Metal–organic frameworks meet scalable and sustainable synthesis. Green Chem. 2017, 19 (12), 2729–2747. 10.1039/C7GC01078H.

[ref23] Gaab M.; Trukhan N.; Maurer S.; Gummaraju R.; Müller U. The progression of Al-based metal-organic frameworks – From academic research to industrial production and applications. Microporous Mesoporous Mater. 2012, 157, 131–136. 10.1016/j.micromeso.2011.08.016.

[ref24] Anastas P.; Eghbali N. Green Chemistry: Principles and Practice. Chem. Soc. Rev. 2010, 39 (1), 301–312. 10.1039/B918763B. 20023854

[ref25] Lin J.-B.; Nguyen T. T. T.; Vaidhyanathan R.; Burner J.; Taylor J. M.; Durekova H.; Akhtar F.; Mah R. K.; Ghaffari-Nik O.; Marx S.; et al. A scalable metal-organic framework as a durable physisorbent for carbon dioxide capture. Science 2021, 374 (6574), 1464–1469. 10.1126/science.abi7281. 34914501

[ref26] Friščić T.; Mottillo C.; Titi H. M. Mechanochemistry for Synthesis. Angew. Chem., Int. Ed. 2020, 59 (3), 1018–1029. 10.1002/anie.201906755. 31294885

[ref27] Tanaka K.; Toda F. Solvent-Free Organic Synthesis. Chem. Rev. 2000, 100 (3), 1025–1074. 10.1021/cr940089p. 11749257

[ref28] Crawford D.; Casaban J.; Haydon R.; Giri N.; McNally T.; James S. L. Synthesis by extrusion: Continuous, large-scale preparation of MOFs using little or no solvent. Chem. Sci. 2015, 6 (3), 1645–1649. 10.1039/C4SC03217A. 29308131 PMC5639793

[ref29] McNaught A.; Wilkinson A. Compendium of chemical terminology, IUPAC Gold, 2nd ed.; 1997.

[ref30] James S. L.; Adams C. J.; Bolm C.; Braga D.; Collier P.; Friščić T.; Grepioni F.; Harris K. D. M.; Hyett G.; Jones W.; et al. Mechanochemistry: Opportunities for new and cleaner synthesis. Chem. Soc. Rev. 2012, 41 (1), 413–447. 10.1039/C1CS15171A. 21892512

[ref31] Shan N.; Toda F.; Jones W. Mechanochemistry and co-crystal formation: Effect of solvent on reaction kinetics. Chem. Commun. 2002, 20, 2372–2373. 10.1039/b207369m. 12430446

[ref32] Strobridge F. C.; Judaš N.; Friščić T. A stepwise mechanism and the role of water in the liquid-assisted grinding synthesis of metal–organic materials. CrystEngComm 2010, 12 (8), 2409 10.1039/c003521a.

[ref33] Xin Y.; Yuan J. Schiff’s base as a stimuli-responsive linker in polymer chemistry. Polym. Chem. 2012, 3 (11), 3045 10.1039/c2py20290e.

[ref34] Sanii R.; Bajpai A.; Patyk-Kaźmierczak E.; Zaworotko M. J. High Yield Low-Waste Synthesis of a Family of Pyridyl and Imidazolyl-Substituted Schiff Base Linker Ligands. ACS Sustainable Chem. Eng. 2018, 6 (11), 14589–14598. 10.1021/acssuschemeng.8b03204.

[ref35] Ghosh M. K.; Pathak S.; Ghorai T. K. Synthesis of Two Mononuclear Schiff Base Metal (M = Fe, Cu) Complexes: MOF Structure, Dye Degradation, H2O2 Sensing, and DNA Binding Property. ACS Omega 2019, 4 (14), 16068–16079. 10.1021/acsomega.9b02268. 31592474 PMC6777120

[ref36] Liu Y.; Yang A.-A.; Zhang X.-S.; Sun Z.-B.; Li W.-Z.; Wang Y.; Luan J.; Liu H.-C. Synthesis of metal–organic coordination polymers and their derived nanostructures for organic dye removal and analyte detection. J. Environ. Chem. Eng. 2022, 10 (4), 108215 10.1016/j.jece.2022.108215.

[ref37] Liu B.; Yuan Q. Two novel linear arrangement d10 hexamers with isonicotinic acid: Structures, blue luminescent and semiconducting properties. Inorg. Chem. Commun. 2005, 8 (11), 1022–1024. 10.1016/j.inoche.2005.07.019.

[ref38] Nandi S.; Collins S.; Chakraborty D.; Banerjee D.; Thallapally P. K.; Woo T. K.; Vaidhyanathan R. Ultralow Parasitic Energy for Postcombustion CO2 Capture Realized in a Nickel Isonicotinate Metal–Organic Framework with Excellent Moisture Stability. J. Am. Chem. Soc. 2017, 139 (5), 1734–1737. 10.1021/jacs.6b10455. 28107782

[ref39] Zhou L.; Fan H.; Zhou B.; Cui Z.; Qin B.; Zhang X.; Li W.; Zhang J. Tetranuclear cobalt(ii)–isonicotinic acid frameworks: Selective CO2 capture, magnetic properties, and derived “Co3O4” exhibiting high performance in lithium ion batteries. Dalton Trans. 2019, 48 (1), 296–303. 10.1039/C8DT04054K. 30516197

[ref40] Kumar N.; Wang S.-Q.; Mukherjee S.; Bezrukov A. A.; Patyk-Kaźmierczak E.; O’Nolan D.; Kumar A.; Yu M.-H.; Chang Z.; Bu X.-H.; et al. Crystal engineering of a rectangular sql coordination network to enable xylenes selectivity over ethylbenzene. Chem. Sci. 2020, 11 (26), 6889–6895. 10.1039/D0SC02123G. 33033602 PMC7500086

[ref41] Fang Z.-L.; Nie Q.-X. Zinc(II) and cadmium(II) complexes of Schiff bases derived from amino acids and pyridine-3-carboxaldehyde: Synthesis, crystal structures, and fluorescence. J. Coord. Chem. 2010, 63 (13), 2328–2336. 10.1080/00958972.2010.500664.

[ref42] Laudert D.; Hohmann H. P. 3.50 - Application of Enzymes and Microbes for the Industrial Production of Vitamins and Vitamin-Like Compounds. In Comprehensive Biotechnology. Moo-Young M. Ed.; Pergamon, 2011, pp. 616–634.

[ref43] Andaloussi Y. H.; Bezrukov A. A.; Sensharma D.; Zaworotko M. J. Supramolecular isomerism and structural flexibility in coordination networks sustained by cadmium rod building blocks. CrystEngcomm 2023, 25 (29), 4175–4181. 10.1039/D3CE00557G. 37492238 PMC10364239

[ref44] Lin W.; Evans O. R.; Xiong R.-G.; Xiong A.; Wang Z. Supramolecular Engineering of Chiral and Acentric 2D Networks. Synthesis, Structures, and Second-Order Nonlinear Optical Properties of Bis(nicotinato)zinc and Bis{3-[2-(4-pyridyl)ethenyl]benzoato}cadmium. J. Am. Chem. Soc. 1998, 120, 13272–13273. 10.1021/ja983415h.

[ref45] Wang H.; Warren M.; Jagiello J.; Jensen S.; Ghose S. K.; Tan K.; Yu L.; Emge T. J.; Thonhauser T.; Li J. Crystallizing Atomic Xenon in a Flexible MOF to Probe and Understand Its Temperature-Dependent Breathing Behavior and Unusual Gas Adsorption Phenomenon. J. Am. Chem. Soc. 2020, 142 (47), 20088–20097. 10.1021/jacs.0c09475. 33172264

[ref46] Li X.; Sensharma D.; Loots L.; Geng S.; Nikkhah S. J.; Lin E.; Bon V.; Liu W.; Wang Z.; He T.; et al. Reversible Phase Transformations in a Double-Walled Diamondoid Coordination Network with a Stepped Isotherm for Methane. J. Am. Chem. Soc. 2024, 146 (27), 18387–18395. 10.1021/jacs.4c03555. 38904843 PMC11240251

[ref47] Subanbekova A.; Bezrukov A. A.; Bon V.; Nikolayenko V. I.; Koupepidou K.; Sensharma D.; Javan Nikkhah S.; Wang S.-Q.; Kaskel S.; Vandichel M.; et al. Effect of Polymorphism on the Sorption Properties of a Flexible Square-Lattice Topology Coordination Network. ACS Appl. Mater. Interfaces 2024, 16 (18), 24132–24140. 10.1021/acsami.4c03777. 38666365 PMC11082895

[ref48] Yang Q. Y.; Lama P.; Sen S.; Lusi M.; Chen K. J.; Gao W. Y.; Shivanna M.; Pham T.; Hosono N.; Kusaka S.; et al. Reversible Switching between Highly Porous and Nonporous Phases of an Interpenetrated Diamondoid Coordination Network That Exhibits Gate-Opening at Methane Storage Pressures. Angew. Chem., Int. Ed. 2018, 57 (20), 5684–5689. 10.1002/anie.201800820. 29575465

[ref49] Subanbekova A.; Nikolayenko V. I.; Bezrukov A. A.; Sensharma D.; Kumar N.; O’Hearn D. J.; Bon V.; Wang S.-Q.; Koupepidou K.; Darwish S.; et al. Water vapour and gas induced phase transformations in an 8-fold interpenetrated diamondoid metal–organic framework. J. Mater. Chem. A 2023, 11 (17), 9691–9699. 10.1039/D3TA01574B. PMC10153660 37153821

[ref50] Groom C. R.; Bruno I. J.; Lightfoot M. P.; Ward S. C. The Cambridge Structural Database. Acta Crystallogr., Sect. B: Struct. Sci., Cryst. Eng. Mater. 2016, 72 (2), 171–179. 10.1107/S2052520616003954. PMC4822653 27048719

[ref51] Shattock T. R.; Arora K. K.; Vishweshwar P.; Zaworotko M. J. Hierarchy of Supramolecular Synthons: Persistent Carboxylic Acid···Pyridine Hydrogen Bonds in Cocrystals That also Contain a Hydroxyl Moiety. Cryst. Growth Des. 2008, 8 (12), 4533–4545. 10.1021/cg800565a.

[ref52] Almarsson Ö.; Zaworotko M. J. Crystal engineering of the composition of pharmaceutical phases. Do pharmaceutical co-crystals represent a new path to improved medicines?. Chem. Commun. 2004, (17), 1889–1896. 10.1039/b402150a. 15340589

[ref53] Nangia A. K.; Desiraju G. R. Heterosynthons Solid Form Design and Enhanced Drug Bioavailability. Angew. Chem., Int. Ed. 2022, 61 (39), e202207484 10.1002/anie.202207484. 35984673

[ref54] Etter M. C.; MacDonald J. C.; Bernstein J. Graph-set analysis of hydrogen-bond patterns in organic crystals. Acta Crystallogr. B 1990, 46 (2), 256–262. 10.1107/s0108768189012929. 2344397

[ref55] Yang J.; Ma J.-F.; Liu Y.-Y.; Ma J.-C.; Batten S. R. A Series of Cu(II) Complexes Based on Different Bis(imidazole) Ligands and Organic Acids: Formation of Water Clusters and Fixation of Atmospheric Carbon Dioxide. Cryst. Growth Des. 2008, 8 (12), 4383–4393. 10.1021/cg701119g.

[ref56] Liu J.; Huang J.; Zhang M.-M.; KongYang Z.-L.; Liang Q.-R.; Chen S.-S. Two Cu(II) microporous frameworks based on a bifunctional linker and selective gas adsorption properties for CO2. J. Solid State Chem. 2023, 318, 123737 10.1016/j.jssc.2022.123737.

[ref57] Luan X.-J.; Wang Y.-Y.; Li D.-S.; Liu P.; Hu H.-M.; Shi Q.-Z.; Peng S.-M. Self-Assembly of an Interlaced Triple-Stranded Molecular Braid with an Unprecedented Topology through Hydrogen-Bonding Interactions. Angew. Chem., Int. Ed. 2005, 44 (25), 3864–3867. 10.1002/anie.200500744. 15892030

[ref58] Shen J.; He X.; Ke T.; Krishna R.; van Baten J. M.; Chen R.; Bao Z.; Xing H.; Dincǎ M.; Zhang Z.; et al. Simultaneous interlayer and intralayer space control in two-dimensional metal–organic frameworks for acetylene/ethylene separation. Nat. Commun. 2020, 11 (1), 6259 10.1038/s41467-020-20101-7. 33288766 PMC7721749

[ref59] Shivanna M.; Otake K.-I.; Song B.-Q.; van Wyk L. M.; Yang Q.-Y.; Kumar N.; Feldmann W. K.; Pham T.; Suepaul S.; Space B.; et al. Benchmark Acetylene Binding Affinity and Separation through Induced Fit in a Flexible Hybrid Ultramicroporous Material. Angew. Chem., Int. Ed. 2021, 60 (37), 20383–20390. 10.1002/anie.202106263. PMC8457195 34250717

[ref60] Gao M.-Y.; Bezrukov A. A.; Song B.-Q.; He M.; Nikkhah S. J.; Wang S.-Q.; Kumar N.; Darwish S.; Sensharma D.; Deng C.; et al. Highly Productive C3H4/C3H6 Trace Separation by a Packing Polymorph of a Layered Hybrid Ultramicroporous Material. J. Am. Chem. Soc. 2023, 145 (21), 11837–11845. 10.1021/jacs.3c03505. 37204941 PMC10236493

[ref61] Li B.; Yan Q.-Q.; Yong G.-P. A new porous coordination polymer reveals selective sensing of Fe3+, Cr2O72–, CrO42–, MnO4– and nitrobenzene, and stimuli-responsive luminescence color conversions. J. Mater. Chem. C 2020, 8 (34), 11786 10.1039/C9TC07030C.

