# Supplementary material for: Green Synthesis of a New Schiff Base Linker and Its Use to Prepare Coordination Polymers
Source: Cryst Growth Des. 2024 Dec 25;25(2):444–51. doi: 10.1021/acs.cgd.4c01606 (PMC11740990; doi:10.1021/acs.cgd.4c01606)
Supplement: Supplementary file 1 — cg4c01606_si_001.pdf [file cg4c01606_si_001.pdf]

Supplementary Information for

Green synthesis of a new Schiff base linker and its use to prepare coordination polymers

*Maria T. Hayes, Aizhamal Subanbekova, Yassin H. Andaloussi, Alan C. Eaby and Michael J. Zaworotko**

Department of Chemical Sciences, Bernal Institute, University of Limerick, Limerick, V94 T9PX, Republic of Ireland

Table of Contents

1. Synthesis 3
2. Materials and methods 5
3. Figures and Tables 10
4. References 38

1. Synthesis

1.1. Synthesis of 4-PIBZ. In equimolar amounts, 3-amino-4-methylpyridine (93.3 mg, 0.86 mmol) and 4-formylbenzoic acid (129.0 mg, 0.86 mmol) along with a catalytic amount of deionized water (100 µL, 11 mmol) were ground using a mortar and pestle for 15 min. A pale-yellow powder was formed. The sample was heated to 140 °C for 3 h to yield the water-free product. The product was obtained as a pale-yellow powder (97 % yield, 200.2 mg). M.P. 194 °C; FTIR 1627 cm^{-1} ($\nu_{CH=N}$) (Figure S1). A larger scale synthesis was performed *via* ball milling for 2 h, followed by heating to 140 °C for 3 h, yielding the same pale-yellow powder (97 % yield, 2.157 g). 1H NMR (400 MHz, DMSO-d_6) δ 2.31 (s, 3H), 7.29 (d, 1H), 8.08 (m, 4H), 8.25 (s, 1H), 8.32 (d, 1H), 8.66 (s, 1H) (Figure S2).

1.2. 4-PIBZ Single crystal growth. 4-PIBZ (15 mg, 0.06 mmol) was dissolved in 1 mL MeOH and allowed to slowly evaporate over 2 weeks. Plank-shaped crystals were obtained from which SCXRD was performed.

1.3. Preparation of sql-PIBZ-Zn MOF.

1.3.1. Single crystal synthesis. $Zn(NO_3)_2{\cdot}6H_2O$ (0.5 mmol, 148.4 mg) in H_2O (10 mL) was layered over **4-PIBZ** (1.0 mmol, 240 mg), which had been dissolved in MeOH (10 mL) though deprotonation with NaOH until pH 7 was reached. The two layers were separated by 2 mL of MeOH/H_2O (1:1) buffer layer. The obtained yellow single crystals were used for SCXRD analysis.

1.3.2. Bulk synthesis with nitrate. A solution of **4-PIBZ** (2.0 mmol, 480.5 mg) in 10 mL of MeOH was deprotonated with NaOH and then combined with a solution of $Zn(NO_3)_2{\cdot}6H_2O$ (0.5 mmol, 149.7 mg) in 10 mL of H_2O. The mixture was stirred at RT for 24 h. The resulting white powder was collected by filtration and air drying overnight. (62 % yield, 221.6 mg).

1.3.3. Bulk synthesis with acetate. A solution of deprotonated **4-PIBZ** (0.5 mmol, 119.8 mg) in 2.5 mL of MeOH was added to a solution of $Zn(CH_3CO_2)_2{\cdot}2H_2O$ (0.125 mmol, 27.0 mg) in 2.5 mL of H_2O. The mixture was left stirring at RT for 24 hours. A white powder was obtained through filtration and air drying overnight. (60 % yield, 52.9 mg).

1.4. Preparation of 1D-PIBZ-Cu MOM.

1.4.1. Single crystal synthesis. $Cu(NO_3)_2{\cdot}3H_2O$ (0.25 mmol, 60.6 mg) in H_2O (10 mL) was layered over **4-PIBZ** (0.5 mmol, 120 mg), which had been dissolved in MeOH (10 mL) through deprotonation with NaOH until pH 7 was reached. Single crystals of **1D-PIBZ-Cu** were obtained by the same layering procedure as described for **sql-PIBZ-Zn**. Blue crystals of **1D-PIBZ-Cu** were obtained after 2 weeks.

1.4.2. Bulk synthesis with nitrate. $Cu(NO_3)_2{\cdot}3H_2O$ (0.5 mmol, 120.8 mg) was dissolved in H_2O (3.5 mL). **4-PIBZ** (1.0 mmol, 240.0 mg) was dissolved in MeOH (3.5 mL) and deprotonated with NaOH until pH 7 was reached. The two solutions were added together and stirred at room temperature for 24 hours. A blue powder was obtained of **1D-PIBZ-Cu** (90 % yield, 243.5 mg).

1.4.3. Bulk synthesis with acetate. Bulk synthesis of **1D-PIBZ-Cu** were obtained by the same procedure as described for **spl-PIBZ-Cu**, by using $Cu(CH_3CO_2)_2{\cdot}H_2O$ (0.25 mmol, 49.8 mg). A blue powder of **1D-PIBZ-Cu** was obtained (67 % yield, 91.0 mg).

1.5. Preparation of 1D-PIBZ-Cd MOM.

1.5.1. Single-crystal synthesis. Single crystals of **1D-PIBZ-Cd** were obtained by the same procedure as described for **1D-PIBZ-Cu**, by using $Cd(NO_3)_2{\cdot}4H_2O$ (0.25 mmol, 60.6 mg). Yellow crystals of **1D-PIBZ-Cd** were obtained after 6 weeks.

2. Materials and methods

All reagents and solvents were purchased from Sigma Aldrich, Thermoscientific, Fluorochem, or TCI and used without further purification.

2.1. Single Crystal X-ray Diffraction (SCXRD) analysis

Crystal structures of synthesized compounds were determined by single crystal X-ray diffraction (SCXRD) at 150 K with either Cu K_α (λ = 1.540598 Å) (for **4-PIBZ** and **sql-PIBZ-Zn**) or Mo radiation Mo K_α (λ = 0.71073 Å) (for **1D-PIBZ-Cu**). The Bruker D8 Quest fixed-chi diffractometers were equipped with Bruker APEX-II CCD detectors, and the nitrogen-flow Oxford Cryosystem attachment. Unit-cell determination, data reduction, and absorption correction (multiscan method) were conducted using the Bruker APEX4 suite with the implemented SADABS software.[1, 2] The SHELX-2018 program package, implemented in OLEX2 v1.5,[3] was used for structure solution and refinement. Structures were solved using the intrinsic phasing method (SHELXT)[4] and refined with SHELXL[5] using the least-squares method. A larger scale synthesis of the linker was performed in a Retsch MM400 ball mill using two 10 mm stainless steel balls at 25 Hz.

2.2. Ball mill synthesis

For upscaled synthesis of **4-PIBZ** a Retsch MM400 ball mill was used using a 10 mL Teflon vessel with two 10 mm plastic balls at 25 Hz.

2.3. Thermogravimetric Analysis (TGA) and Differential Scanning Calorimetry (DSC)

Thermogravimetric Analysis (TGA) was performed on a TA Instrument Q50 instrument. Around 10 mg of the sample was placed in an aluminum pan and heated at rate of 10 °C min^{-1} from room temperature up to 500 °C under continuous flow of N_2. Differential scanning calorimetry (DSC) analysis was performed on Q2000 TA instrument. Around 5 mg of sample were loaded onto an

aluminum pan and heated at rate of 5 °C min^{-1} from room temperature up to 300 °C under continuous flow of N_2.

2.4.Fourier Transformed Infrared (FTIR) Spectroscopy

Fourier Transform Infrared (FTIR) spectra were collected on a PerkinElmer Spectrum 100 spectrometer with Universal ATR accessory from 750 cm^{-1} to 4000 cm^{-1}.

2.5.Powder X-ray Diffraction (PXRD) analysis

Powder X-ray diffraction data was collected on *ca.* 10 mg of crushed microcrystalline **1D-PIBZ**, **sql-PIBZ-Zn**, and **1D-PIBZ-Cu**. PXRD patterns were recorded using a PANalytical Empyrean diffractometer equipped with a PIXcel3D detector, and operating at 40 kV and 40 mA, and CuKα radiation (λα = 1.540598 Å) was used for diffraction experiments. Incident beam optics included the Fixed Divergences slit 9 with anti-scatter slit PreFIX module, with a 1/8° divergence slit and a 1/4° anti-scatter slit, as well as a 10 mm fixed incident beam mask and a Soller slit (0.04 rad). Divergent beam optics included a P7.5 anti-scatter slit, a Soller slit (0.04 rad), and a Ni-β filter. The data was collected from 5°-40° (2θ) with a step-size of 0.016413° and a varied scan time of 10 seconds per step. Calculated PXRD patterns were calculated from SCXRD structures extracted from Mercury.

2.6.Variable Temperature Powder X-ray Diffraction (VTPXRD) analysis

PXRD patterns at different temperatures were recorded using a PANalytical X'Pert Pro-MPD diffractometer equipped with a PIXcel3D detector operating in scanning line detector mode. An Anton Paar TTK 450 stage coupled with the Anton Paar TCU 110 Temperature Control Unit was used to record the variable temperature diffractograms. The diffractometer was operated at 40 kV and 40 mA and CuKα radiation (λα = 1.540598 Å). Incident beam optics included Fixed Divergences slits, with a 1/4° divergence slit and a Soller slit (0.04 rad). Divergent beam optics

included a P7.5 S7 anti-scatter slit, a Soller slit (0.04 rad), and a Ni-β filter. In this experiment, 50 mg of anhydrous **4-PIBZ** was ground into a fine powder and was loaded on a zero background disc made for Anton Paar TTK 450 chamber. The data was collected from 4°-40° (2θ) with a step-size of 0.0167113° and a scan time of 200 seconds per step under continuous flow of N_2.

2.7. Gas Sorption Experiment

The sorption isotherm for CO_2 at 195 K was measured using Micromeritics Tristar II 3030 instrument. Before the sorption experiment the freshly prepared samples of **4-PIBZ**, **sql-PIBZ-Zn**, and **1D-PIBZ-Cu** were activated under dynamic vacuum at 100 °C on Micromeritics SmartVacPrep instrument for overnight to isolate guest-free phases. The temperature of 195 K was maintained by using 4 L dewar filled with dry ice-acetone mixture. High purity gas was used as received from BOC Gases Ireland CO_2 (99.995%).

2.8. Dynamic Water Sorption (DVS) experiment

Water vapor sorption isotherm determination was performed using Adventure Dynamic Vapor Sorption (DVS) instrument manufactured by Surface Measurement Systems. The instrument gravimetrically measured water vapor uptake using air as a carrier gas. Digital mass flow controllers regulated the flows of dry and saturated gases. Relative humidity was generated by precisely mixing dry and saturated gas flows in desired flow ratios which produced expected relative humidity. Pure water was used to generate water vapor for these measurements and the temperature was maintained at 300 K by enclosing the system in a temperature-controlled incubator. The mass of the sample was determined by a high-resolution microbalance Ultrabalance Low Mass with a precision of 0.01 μg. The microbalance

had symmetric configuration with two branches of the balance being exposed to the same gas and being kept at the same temperature, which allowed negation of buoyancy and drag effects. A flow of 400 sccm (Standard Cubic Centimetres per Minute) was used for the measurements at 300 K. Prior to the measurement, anhydrous **4-PIBZ** was activated *in situ* in dry air at 373 K for 60 minutes using a built-in preheater and consequently cooled to sorption temperature in 90 minutes. Isotherm measurements were performed on *ca.* 10 mg of activated **4-PIBZ**. For each isotherm point, dm/dt < 0.01 % min-1 was used as criteria of reaching equilibrium.

Table S1. Selected crystallographic data and structure refinement parameters.

Compound	4-PIBZ-MeOH	1D-PIBZ-Cd	1D-PIBZ-Cu	sql-PIBZ-Zn
**Formula**	$C_{14}H_{12}N_2O_2$, 1.5(CH_4O)	($C_{28}H_{26}N_4O_6Cd$)$_2$	$C_{28}H_{22}CuN_4O_4$, $C_{28}H_{26}CuN_4O_6$, 0.696(CH_4O)$_2$	($C_{28}H_{22}N_4O_4Zn$)$_2$, (H_2O)$_{12}$, ($C_8H_6O_3$)
**MW (g·mol^{-1})**	288.32	1253.85	1164.65	1454.05
**T (K)**	150.00	150.00	150.00	150.00
**Crystal system**	Monoclinic	Triclinic	Triclinic	Triclinic
**Space group**	$P2_1/n$	$P\bar{1}$	$P\bar{1}$	$P\bar{1}$
**a (Å)**	3.8256(2)	11.1169(4)	7.3249(3)	11.9324(10)
**b (Å)**	20.3557(9)	11.5782(4)	12.9124(5)	13.1777(12)
**c (Å)**	18.6770(7)	12.4507(4)	14.2164(5)	13.3544(10)
**α (°)**	90	106.072(11)	84.6400(4)	73.892(5)
**β (°)**	93.910(2)	109.464(13)	83.5410(4)	65.055(4)
**γ (°)**	90	104.040(12)	77.7220(4)	63.638(4)
**V (Å^3)**	1451.04(11)	1349.13(8)	1302.17(9)	1695.4(3)
**ρ_{calc} (g·cm^{-3})**	1.320	1.543	1.485	1.424
**Z, Z'**	4, 1	1, 0.5	1, 0.5	1, 0.5
**Observed reflections**	2673	5277	5347	5933
**R_1, wR_2 [I > 2σ(I)]**	0.0787, 0.2384	0.0402, 0.1098	0.0347, 0.0898	0.0941, 0.2943
**R_1, wR_2 (all data)**	0.0971, 0.2603	0.0441, 0.1140	0.0409, 0.0941	0.1059, 0.3052
**Goodness-of-fit on F^2**	1.115	1.062	1.042	1.073
**R_{int} value (%)**	9.20	5.49	4.99	6.97
**CCDC number**	2377992	2377993	2377994	2377995

3. Figures and Tables

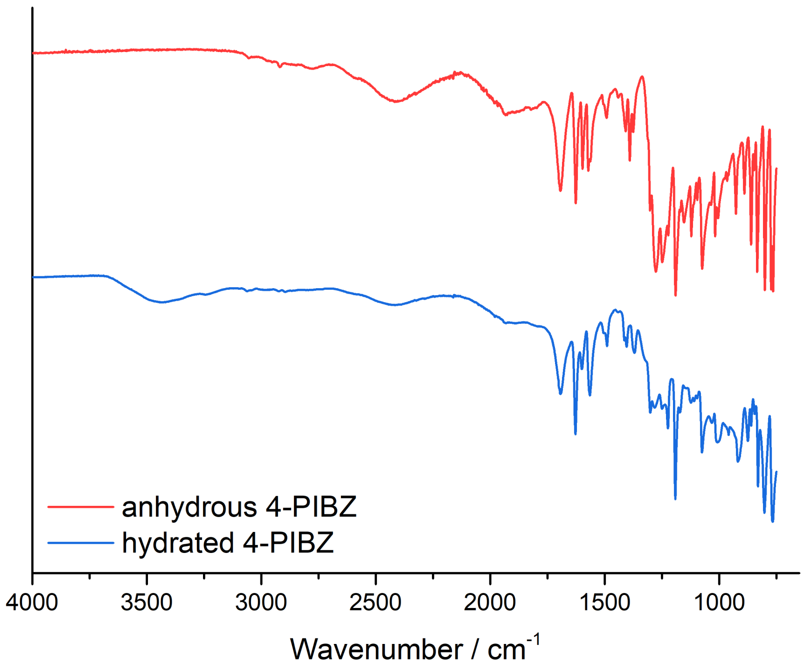

Figure S1. FTIR spectra of **4-PIBZ** hydrated and anhydrous phase.

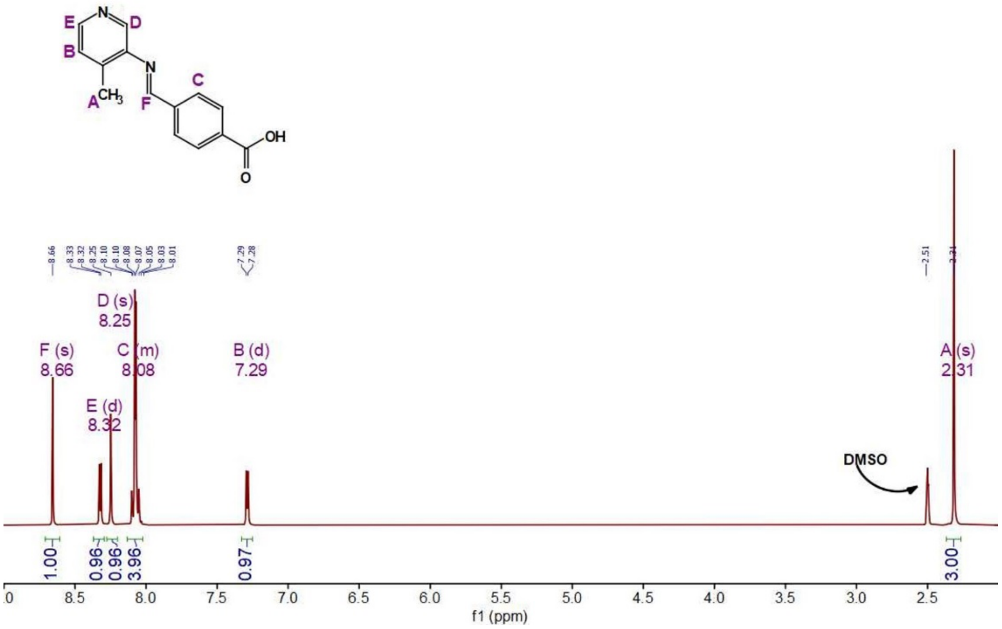

Figure S2. NMR of **4-PIBZ**.

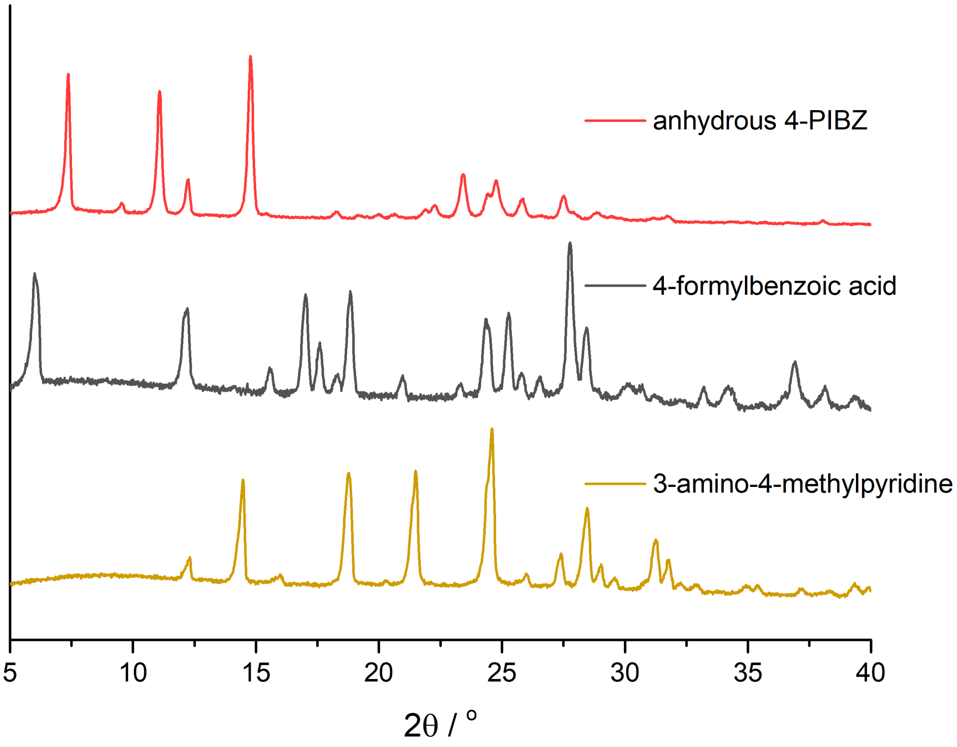

Figure S3. PXRD patterns of anhydrous **4-PIBZ** and its comparison with starting materials.

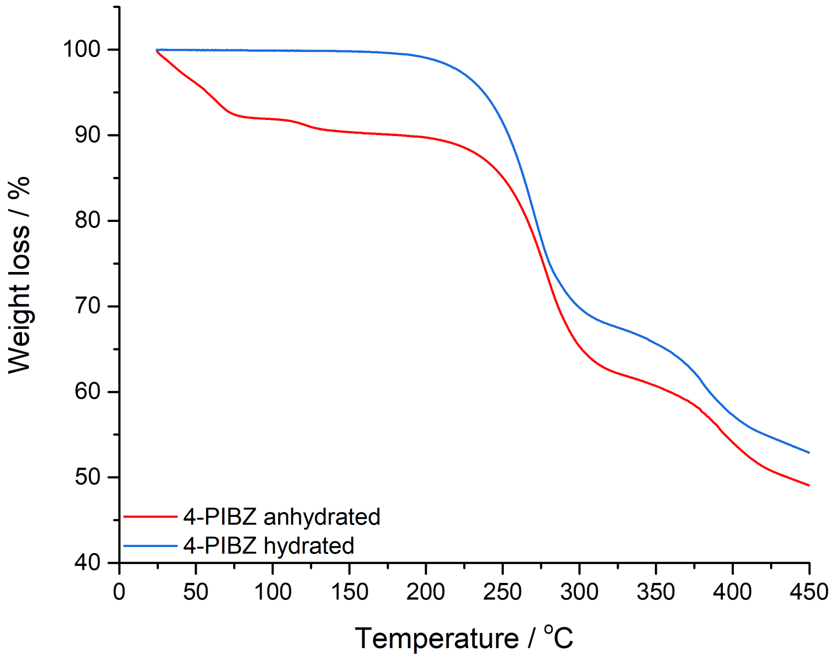

Figure S4. TGA for anhydrous and hydrated **4-PIBZ.**

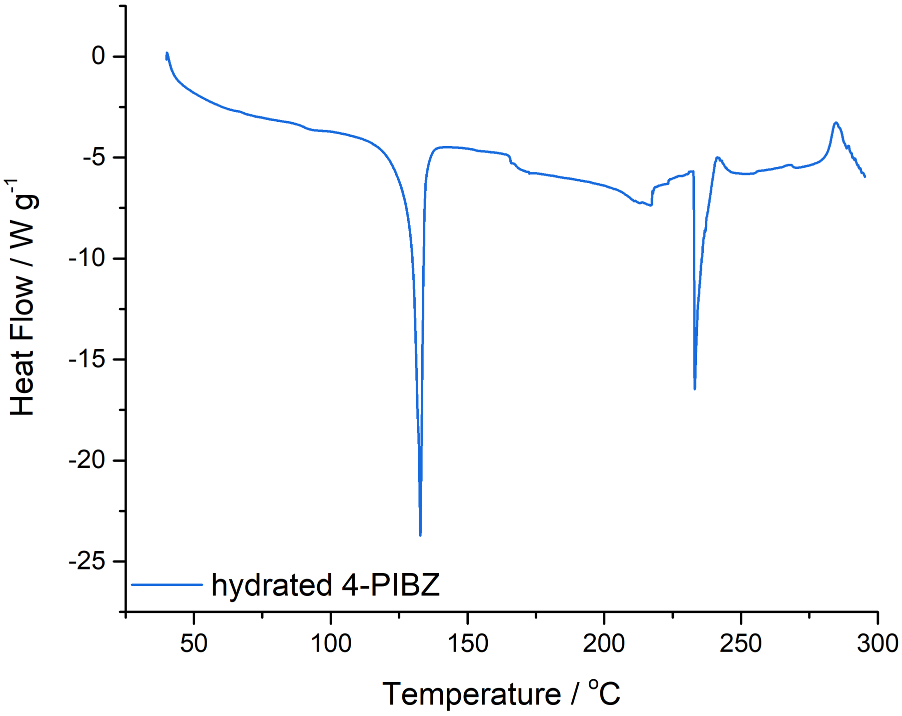

Figure S5. DSC for hydrated **4-PIBZ**.

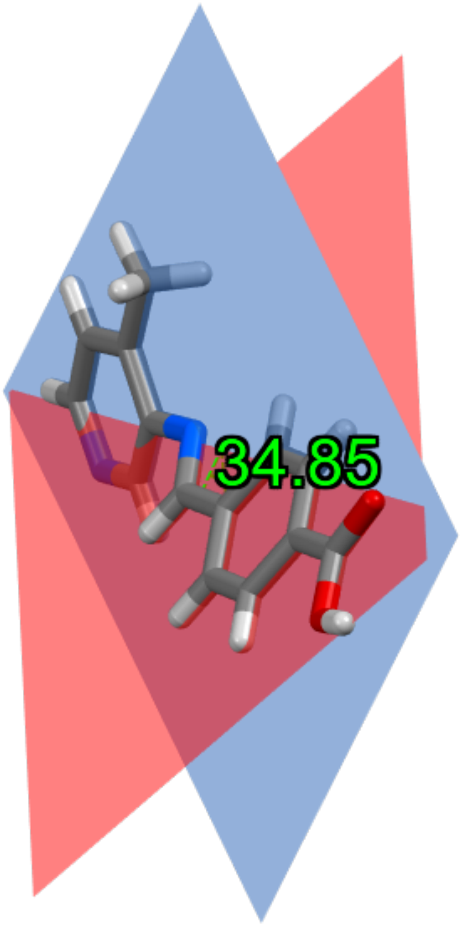

Figure S6. The angle between planes of the pyridyl and phenyl rings.

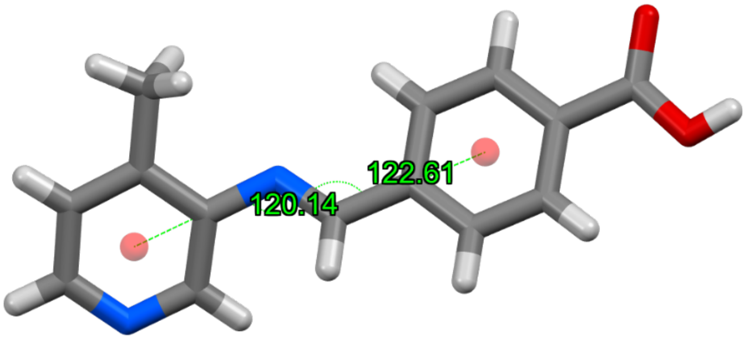

Figure S7. The dihedral angle around imine bond in **4-PIBZ**.

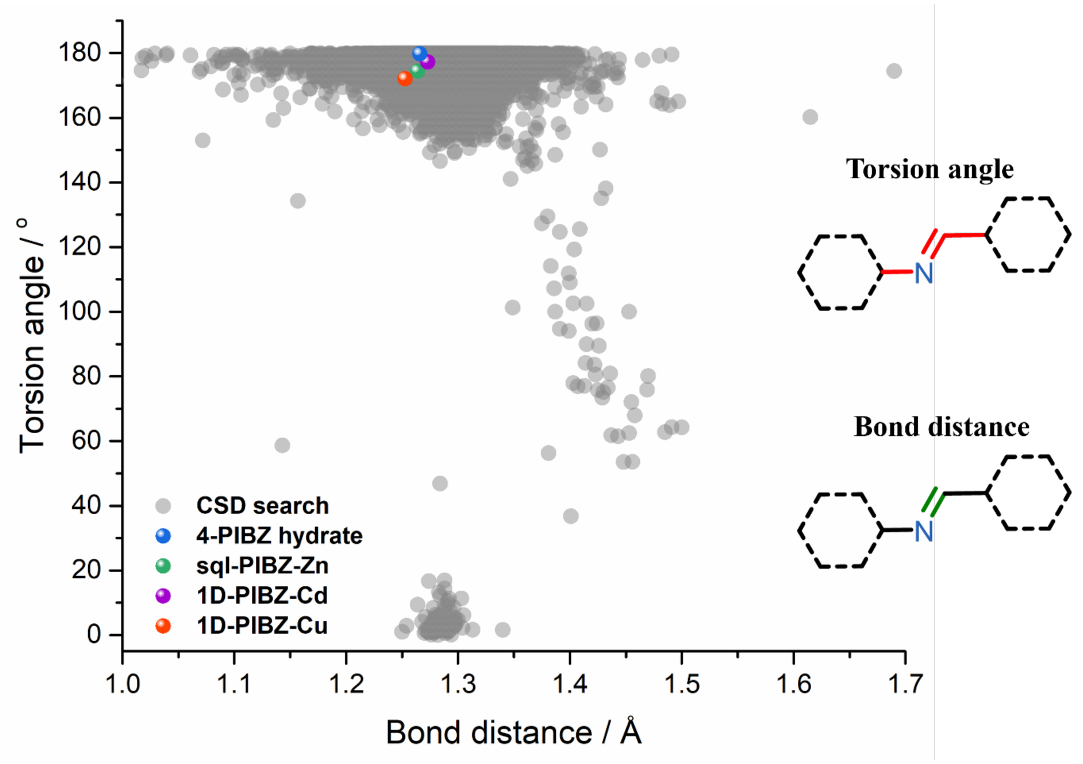

Figure S8. The torsion angle of imine and its bond distance in context with CSD search.

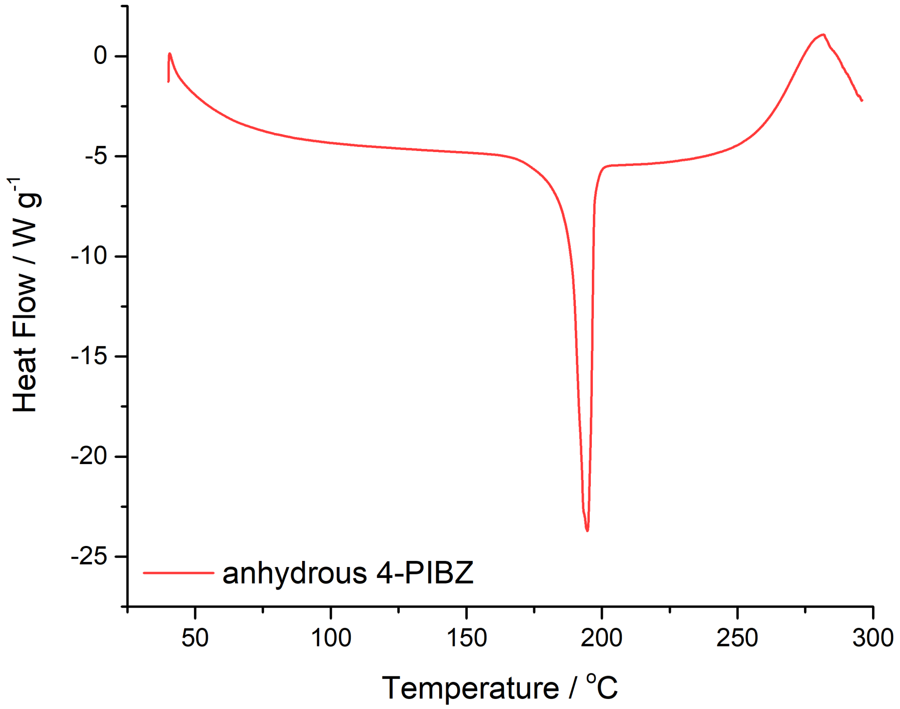

Figure S9. DSC of anhydrous **4-PIBZ**.

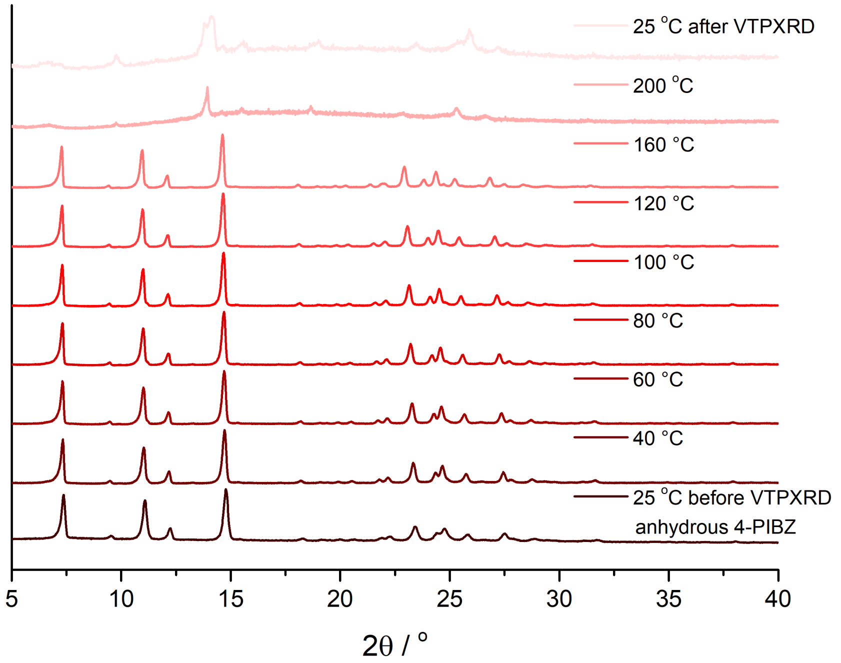

Figure S10. VTPXRD of anhydrous 4-PIBZ heated from RT to 200 °C.

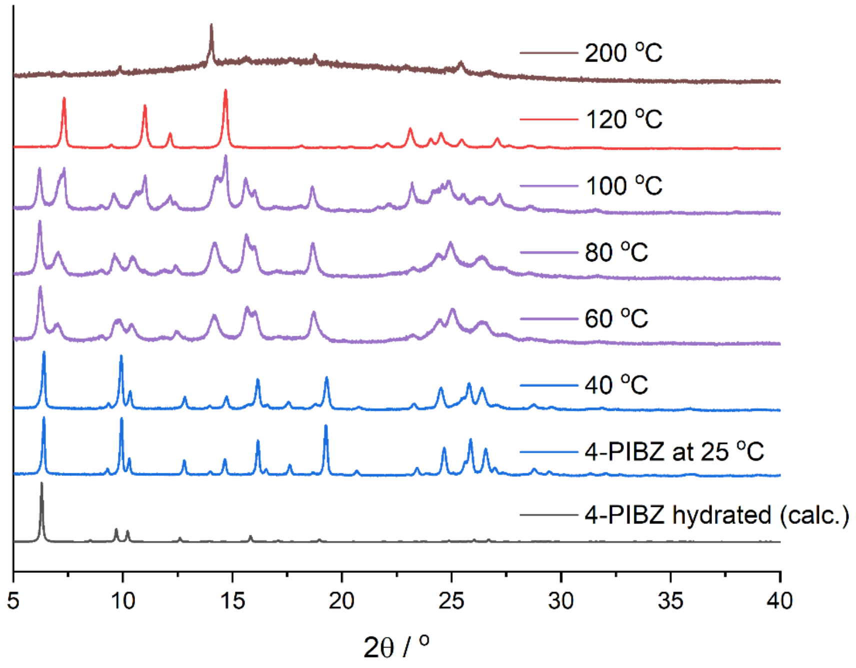

Figure S11. VTPXRD of hydrated **4-PIBZ** starting from RT to 200°C.

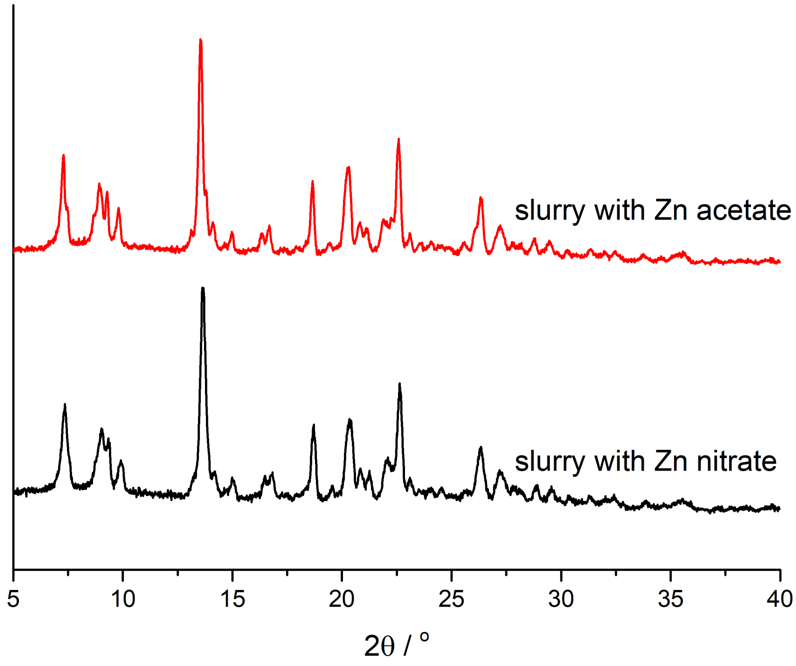

Figure S12. PXRD patterns of **sql-PIBZ-Zn** obtained *via* slurry of zinc nitrate and zinc acetate.

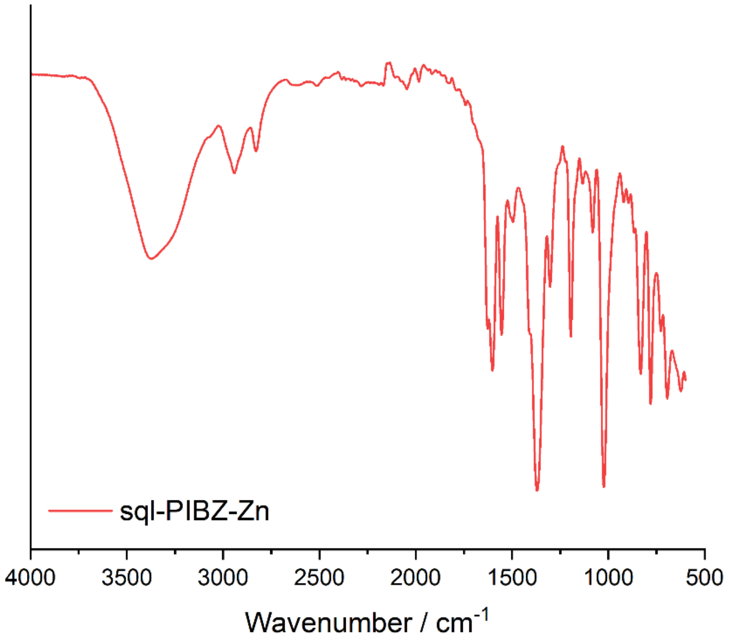

Figure S13. FTIR spectra of as-synthesized **sql-PIBZ-Zn**.

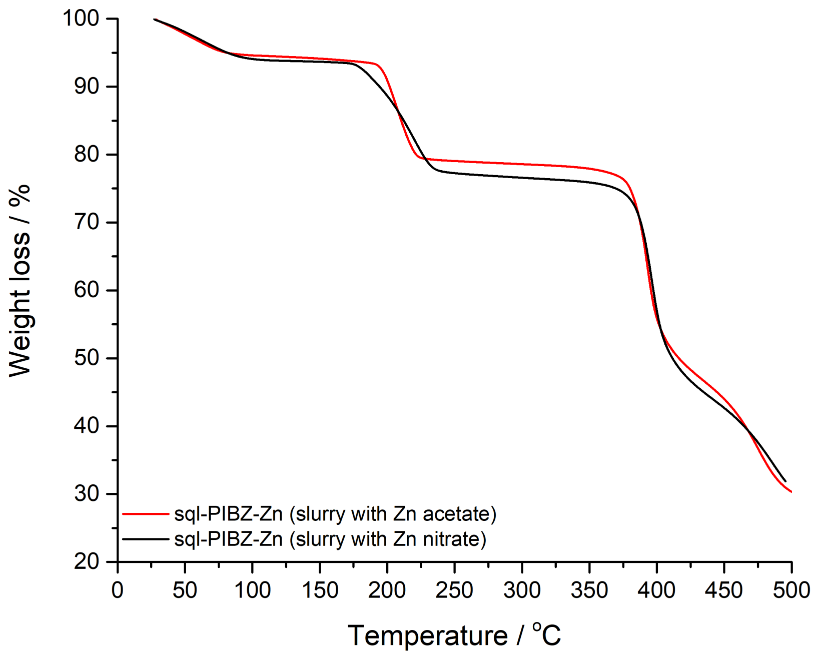

Figure S14. TG analysis of **sql-PIBZ-Zn** obtained via slurry of zinc nitrate and zinc acetate.

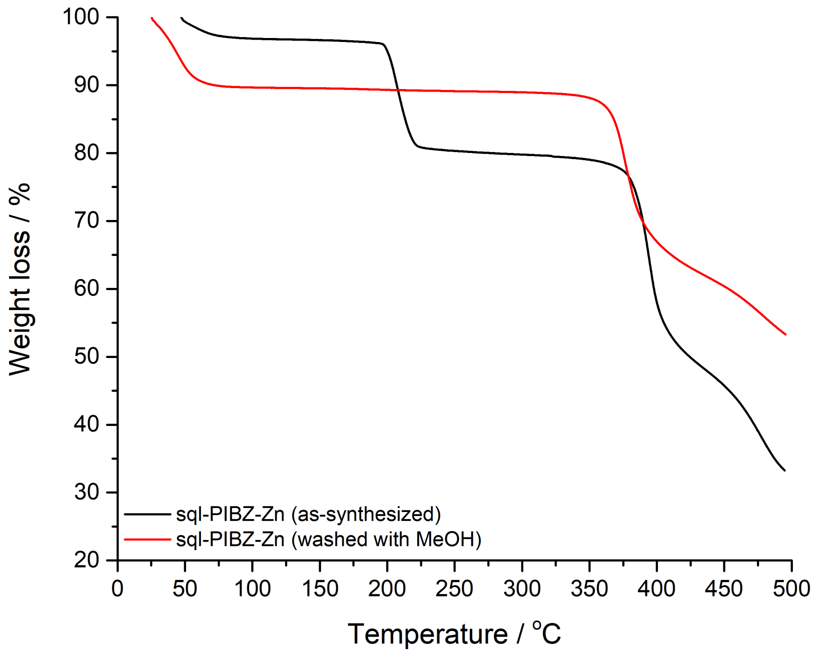

Figure S15. TGA patterns of as-synthesized and washed **sql-PIBZ-Zn**.

a)

b)

c)

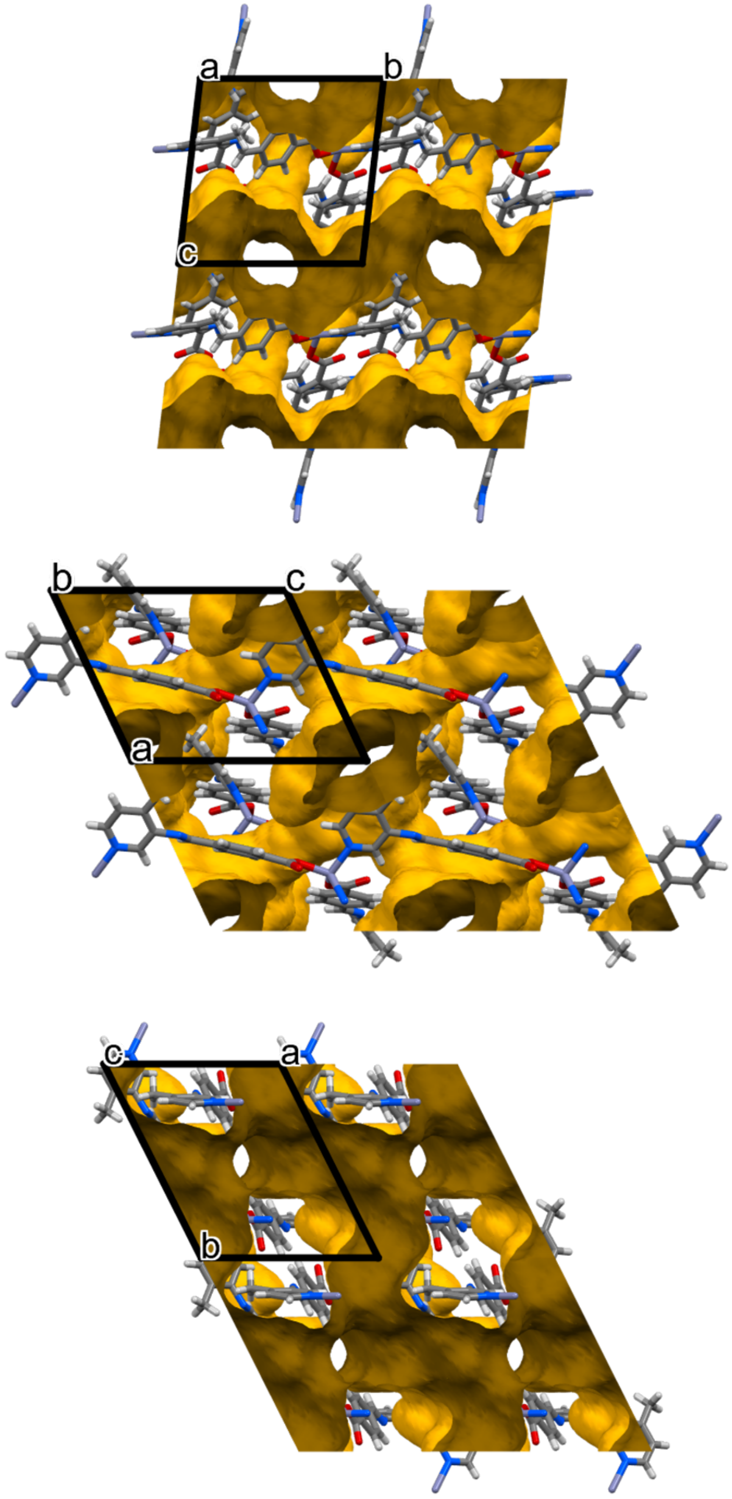

Figure S16. Accessible void space in **sql-PIBZ-Zn** along a) *a*-axis, *b*-axis and c) *c*-axis.

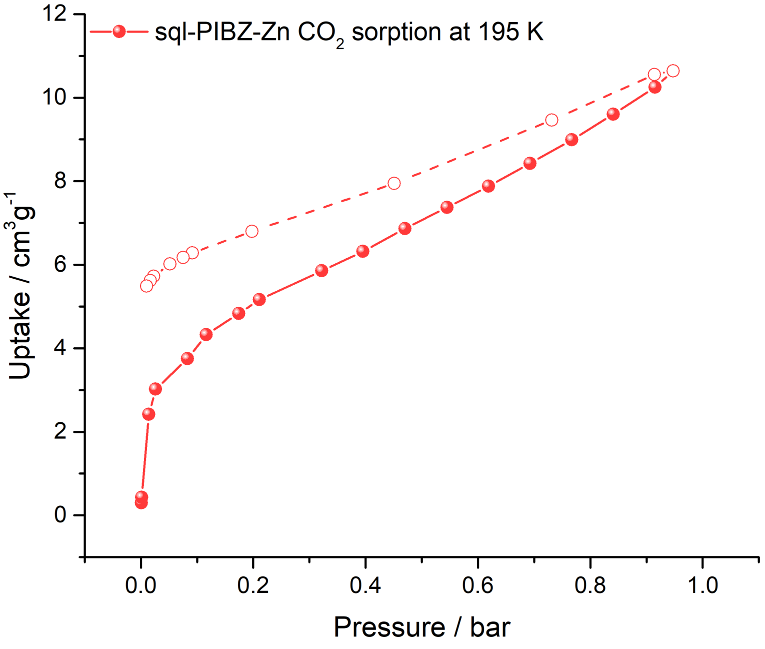

Figure S17. CO_2 sorption isotherm collected at 195 K for **sql-PIBZ-Zn**.

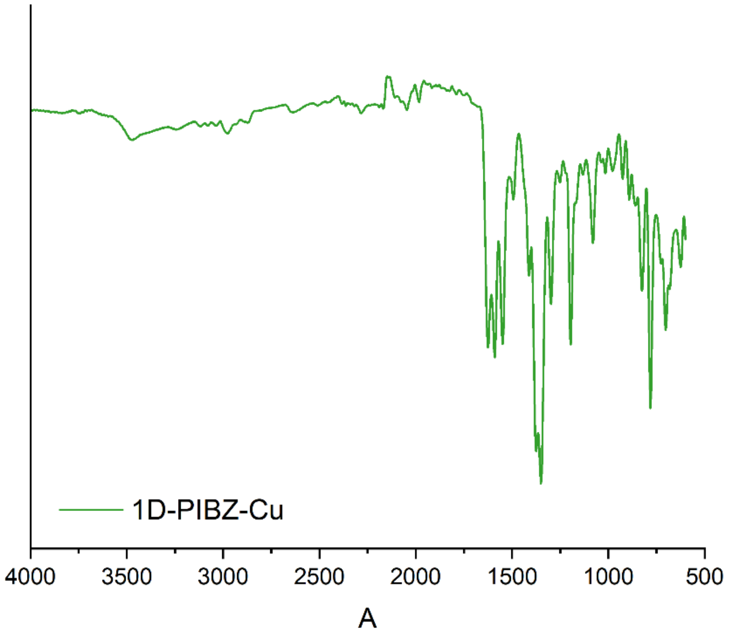

Figure S18. FTIR spectrum of 1D-PIBZ-Cu.

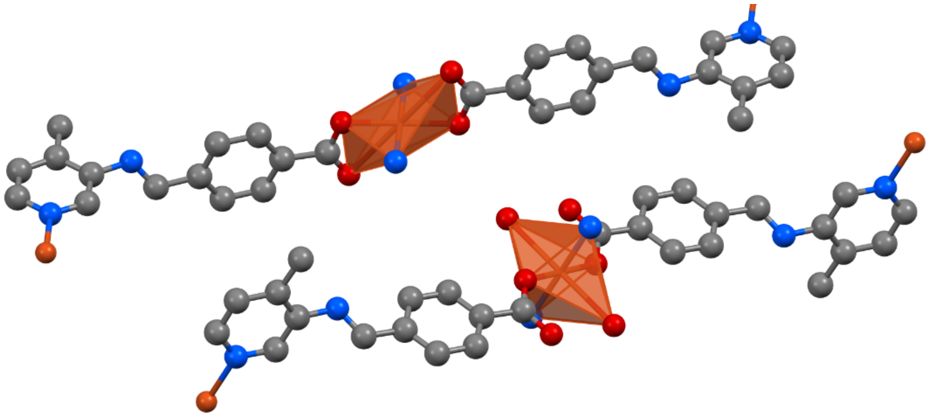

Figure S19. Octahedral and square planar Cu^{2+} environment of two independent chains of **1D-PIBZ-Cu** in asymmetric unit.

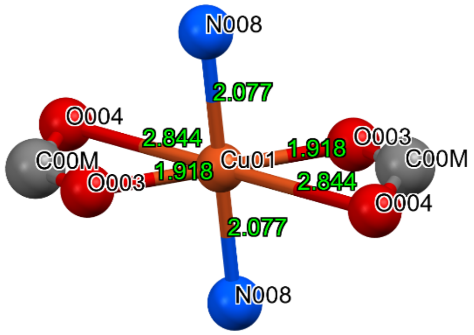

Figure S20. Cu1 environment in **1D-PIBZ-Cu**.

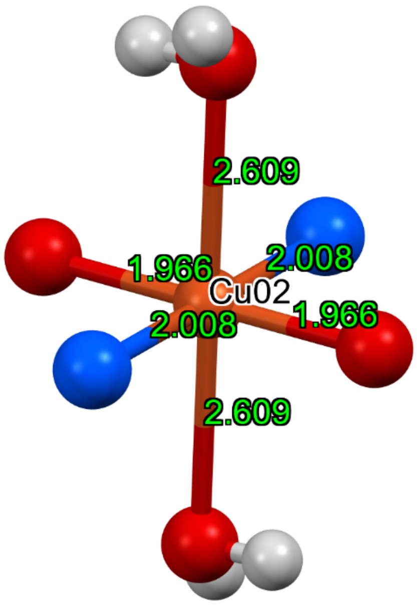

Figure S21. Cu2 environment in **1D-PIBZ-Cu**.

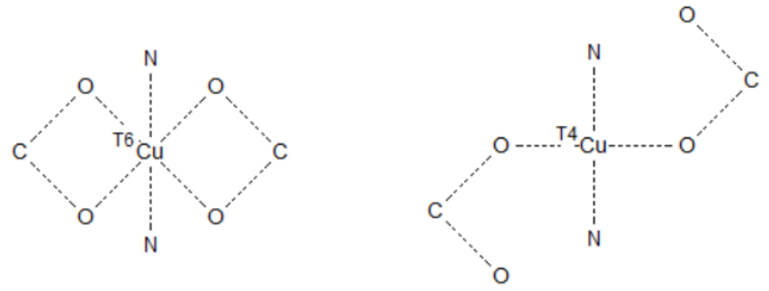

Figure S22. CSD search parameters in ConQuest version 2024.2.0 used to identify known structures with both 4-connected copper atoms (with 2 x μ_1-η^1 carboxylates) and 6-connected copper atoms (with 2 x μ_2-η^1:η^1 carboxylates).

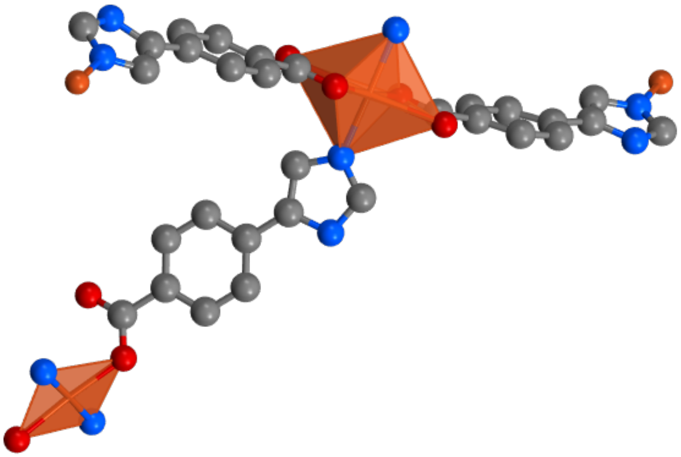

Figure S23. JEQZUE structure featuring different Cu^{2+} environments.

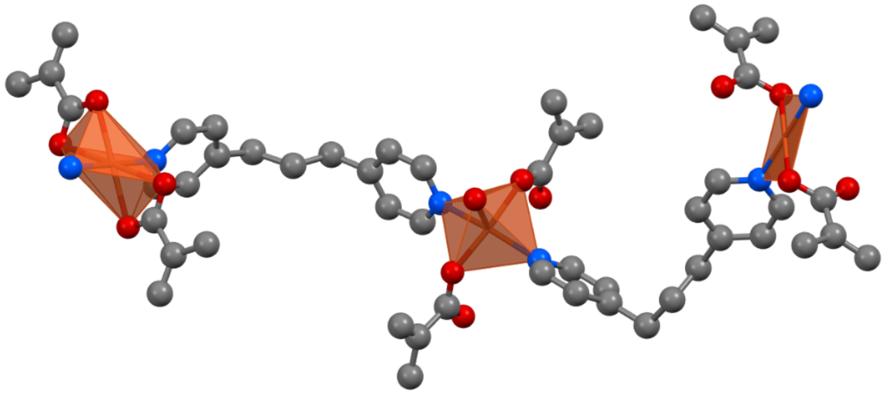

Figure S24. GAQYUT structure featuring different Cu^{2+} environments.

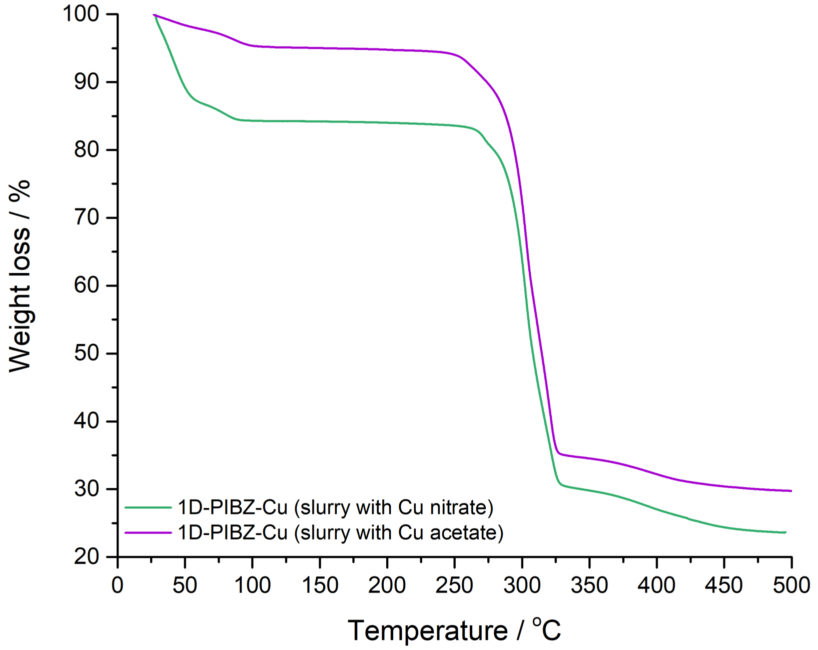

Figure S25. TG analysis of **1D-PIBZ-Cu** obtained via slurry of Cu nitrate and Cu acetate.

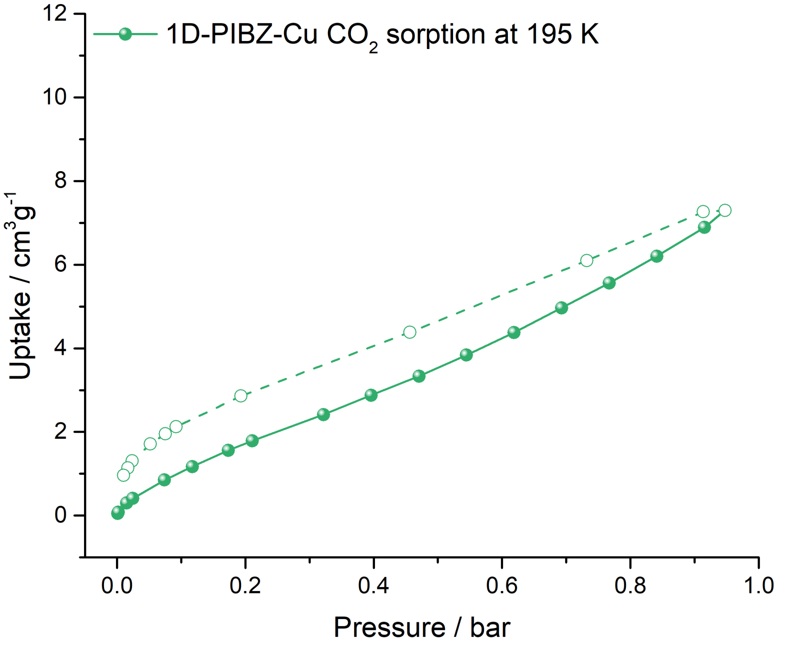

Figure S26. CO_2 sorption isotherm collected at 195 K for **1D-PIBZ-Cu**.

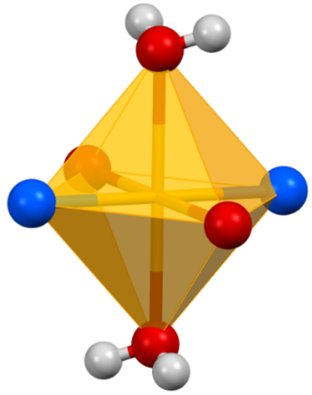

Figure S27. Octahedral Cd^{2+} in **1D-PIBZ-Cd**.

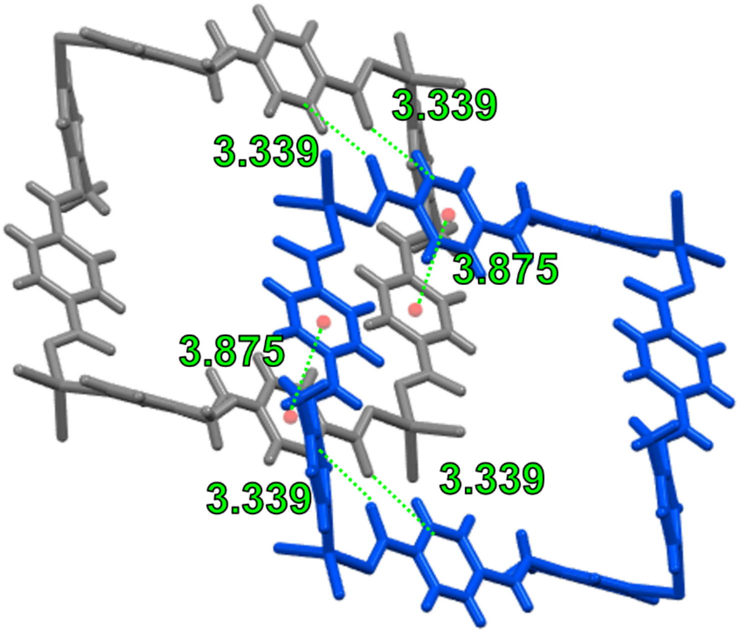

Figure S 28. π- π stacking and short contacts distances between two layers of **sql-PIBZ-Zn**.

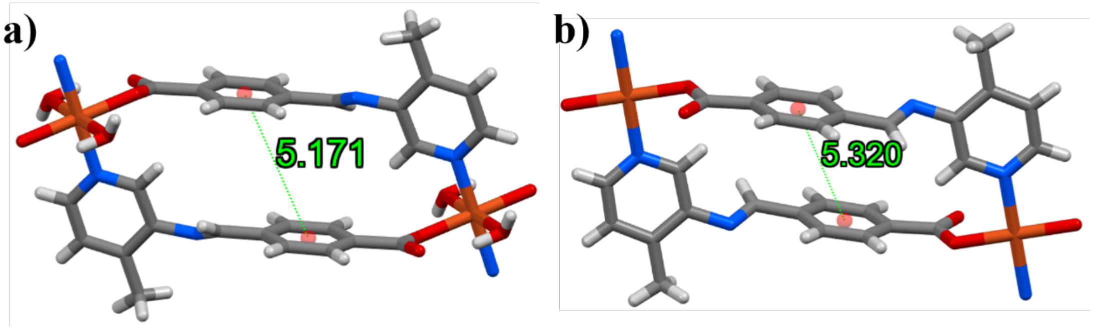

Figure S 29. π- π stacking distance withing spiro loop of two different Cu environment of **1D-PIBZ-Cu** a) octahedral and b) square planar.

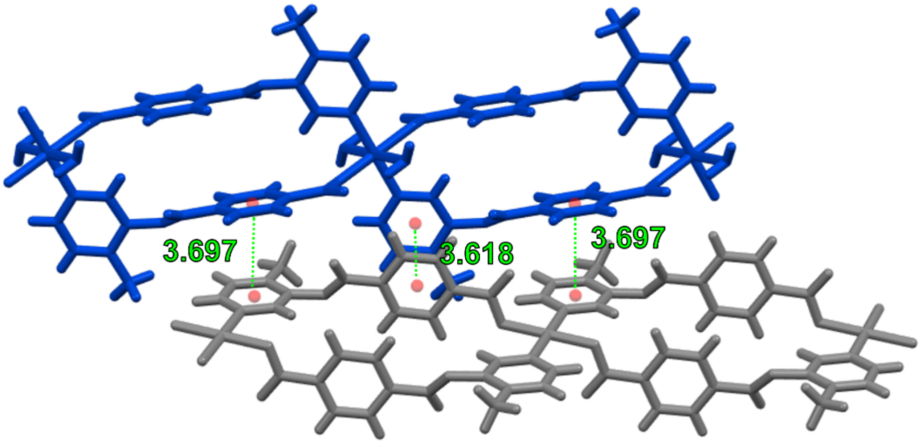

Figure S30. π- π stacking distances between 1D spiro loop chains of **1D-PIBZ-Cu**.

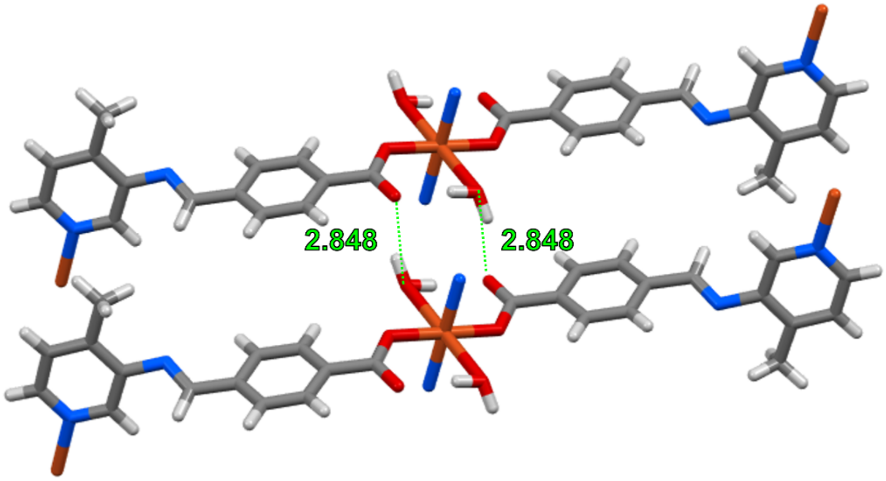

Figure S31. Hydrogen bonding interactions between coordination polymers in **1D-PIBZ-Cu**.

Table S2. Distances between centroids of aromatic rings within the spiro loop of reported structures exhibiting gas uptakes.

No	Ref Code	Linker	Distance / Å	Reference
**1**	AHIDUT	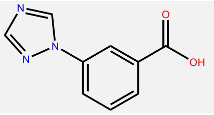	7.062	6
**2**	AMIPUJ	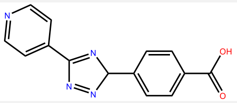	6.291	7
**3**	APAYOG	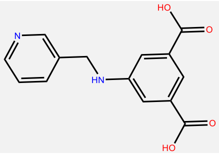	4.125	N/A
**4**	ARUWOB	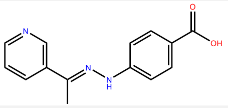	5.402	8
**5**	ARUYAP	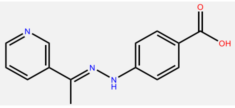	6.700	8
**6**	ASATUK	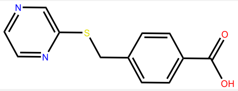	6.394	N/A
**7**	ATETIE	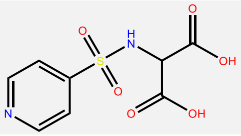	7.106	9
**8**	AXAHEP	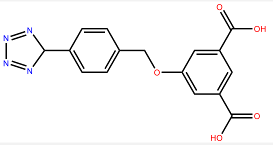	9.309	10
**9**	BANHAA	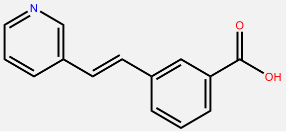	9.065	11
**10**	BEQJIT	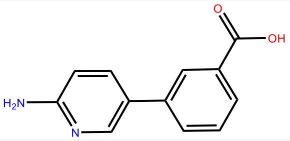	7.012	12
**11**	BOKJES01	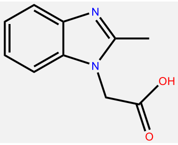	5.113	13
**12**	CAVGEO	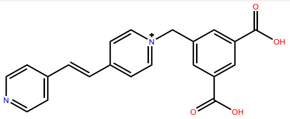	7.723	14

**13**	CECWOZ	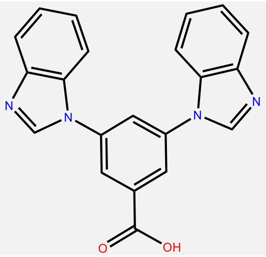	7.868	[15]
**14**	CEMHOV	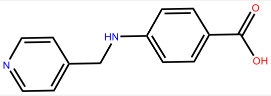	7.381	[16]
**15**	CERKAO	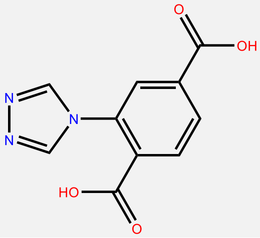	3.980	[17]
**16**	CIRBEL	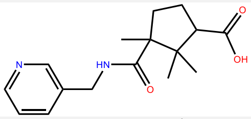	7.549	[18]
**17**	CIBRIP	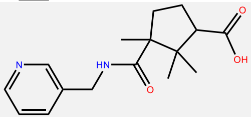	7.548	[18]
**18**	CUSVIW	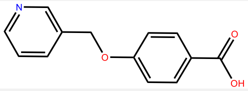	4.916	[19]
**19**	CUTGAB	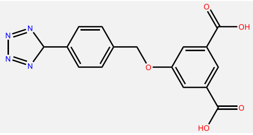	7.493	CSD communication
**20**	CUVHUZ	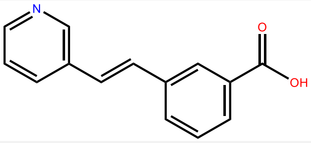	7.529	[20]
**21**	CUZRIZ	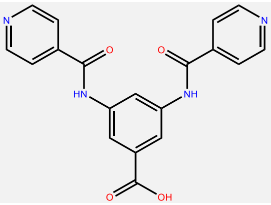	7.948	CSD communication
**22**	DAFYOZ	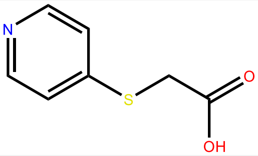	4.761	[21]
**23**	DAMVAS	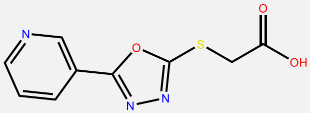	5.067	[22]

**24**	DEBZAP	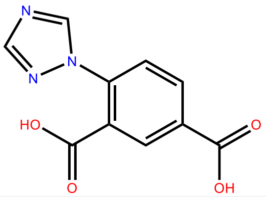	6.478	23
**25**	DIVFIZ	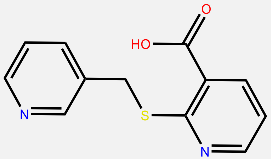	5.772	24
**26**	DOBFAC	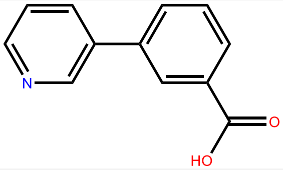	6.837	25
**27**	DOBFEG	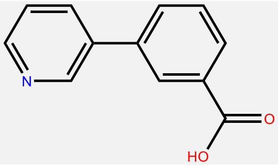	7.640	25
**28**	DOBFIK	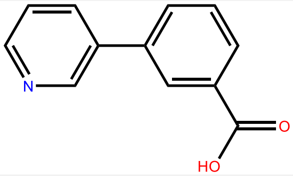	6.900	25
**29**	DOBFOQ	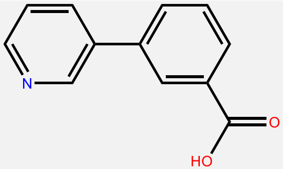	7.461	25
**30**	DOBFUW	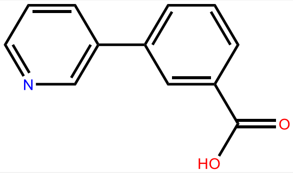	6.928	25
**31**	DOBKIE	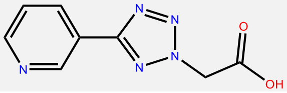	7.248	26
**32**	DOQLUT	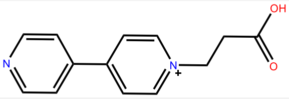	3.995	27
**33**	DOQPAD	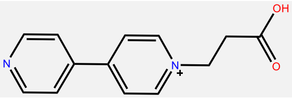	3.738	27
**34**	DOWHII	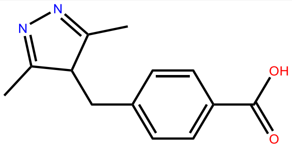	5.900	28

**35**	DUKWUD	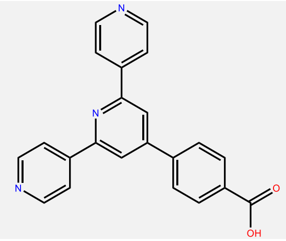	8.917	29
**36**	EJEZID	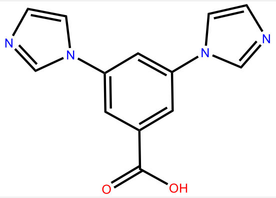	8.431	30
**37**	EJUWOX	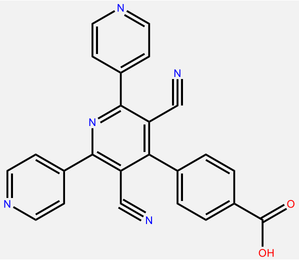	9.774	31
**38**	ETOZOD	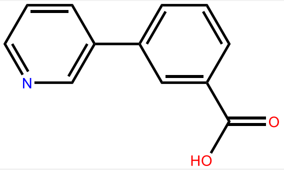	8.144	32
**39**	ETOZOD01	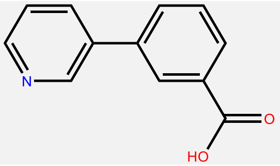	8.157	33
**40**	FECCAV	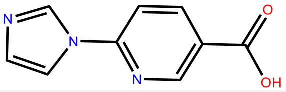	4.023	34
**41**	FEJYID01	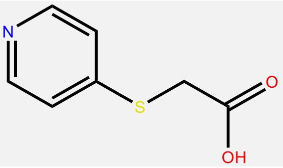	5.067	35
**42**	FEJYID02	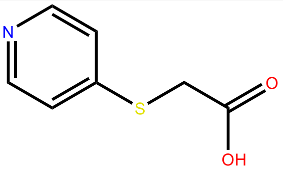	5.226	36

**43**	GEDLAE	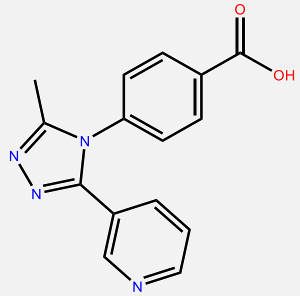	6.332	[37]
**44**	GEKJUE	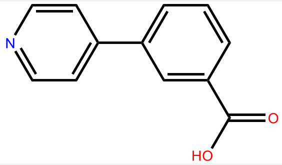	6.178	[38]
**45**	GICDOO	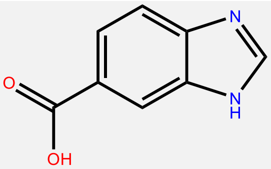	4.435	[39]
**46**	GIGYUR	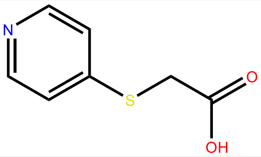	5.532	[40]
**47**	GITFUM	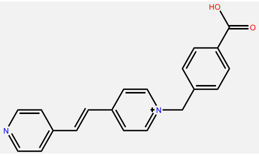	8.908	[41]
**48**	GITGEX	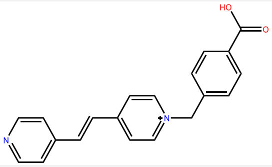	9.140	[41]
**49**	HASQUQ	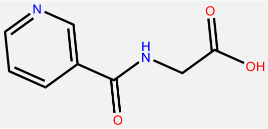	7.428	[42]
**50**	ICOBIN	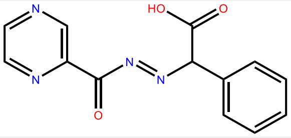	4.399	[43]
**51**	IHIBIM	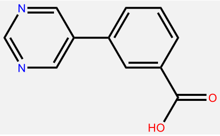	8.245	CSD communications
**52**	IKEXIN	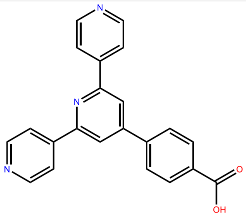	10.562	[44]

**53**	IWATOQ	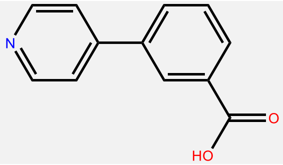	6.157	[45]
**54**	KAGKOV	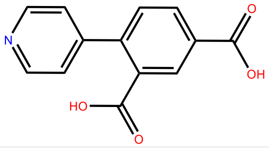	5.274	[46]
**55**	KARDUE	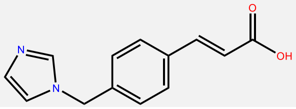	5.633	[47]
**56**	KATKAT	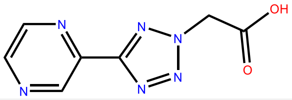	7.186	[48]
**57**	KATKUN	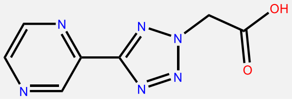	7.105	[48]
**58**	KATLAU	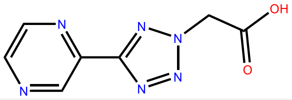	6.795	[48]
**59**	KEPYIO	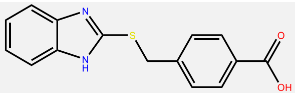	6.071	[49]
**60**	KOHBEP	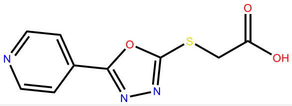	3.748	[50]
**61**	LACCEZ	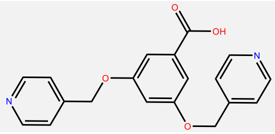	8.007	[51]
**62**	LACCEZ01	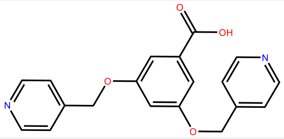	8.002	[52]
**63**	LACCID	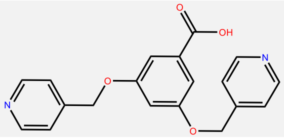	8.026	[51]
**64**	LAGQUI	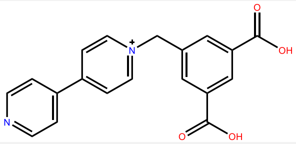	7.492	[53]
**65**	LARZUB	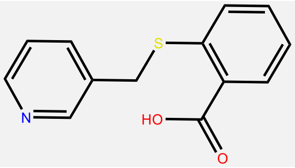	8.538	[54]
**66**	LEGMIW	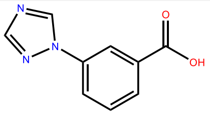	7.297	[55]

**67**	LOTKEM01	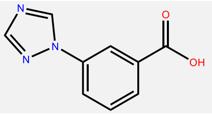	7.932	56
**68**	MAKSIC	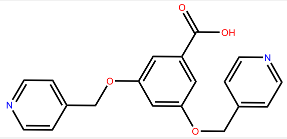	8.457	57
**69**	MICTEA	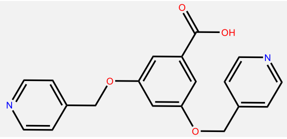		58
**70**	MICTIE	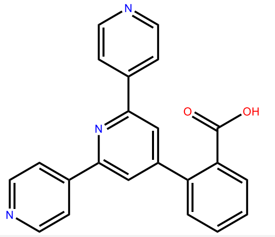		58
**71**	MUGGEB	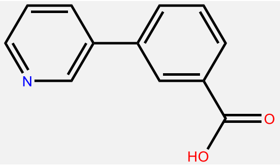	7.998	59
**72**	MUXVOT	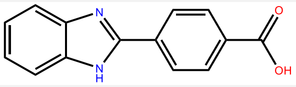	4.093	60
**73**	NEFQUO	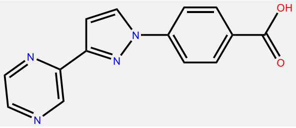	5.882	61
**74**	NENDAL	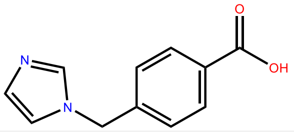	6.339	62
**75**	NIZCEH	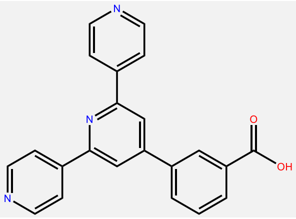	9.583	63
**76**	NOPCIG	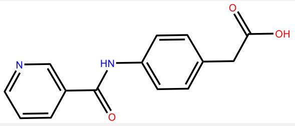	4.611	64
**77**	NOPLEL	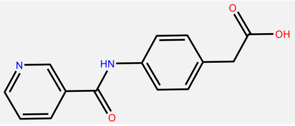	4.700	64
**78**	NOPLUB	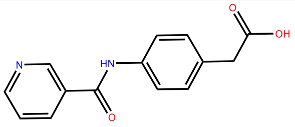	4.642	64

**79**	NUNXUQ	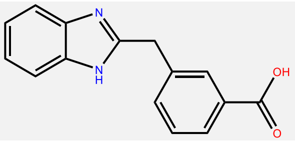	7.167	[65]
**80**	OKOXAP	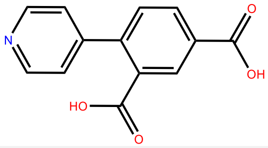	5.492	[66]
**81**	OVEGUS	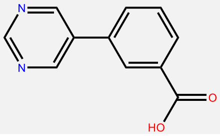	7.467	[67]
**82**	OYOYOR	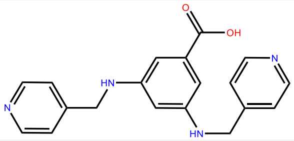	6.480	[68]
**83**	PAYLEI	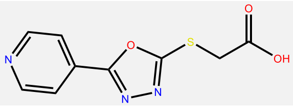	4.127	[69]
**84**	POKXOE	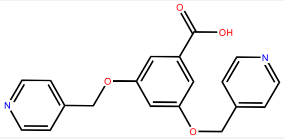	6.734	[70]
**85**	POLCAW	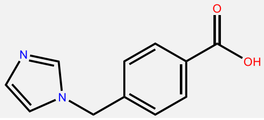	5.617	[71]
**86**	POSGIP	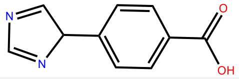	4.086	[72]
**87**	PUZFEY	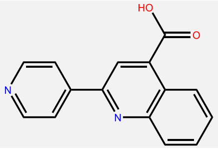	5.429	[73]
**88**	QEBMIW	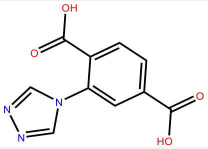	4.170	[74]
**89**	QOMBIG	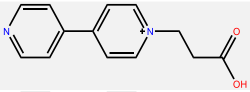	4.772	[75]
**90**	QOMBOM	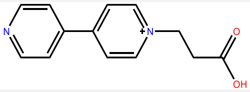	4.008	[75]
**91**	REDVAB	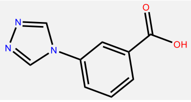	7.692	[76]
**92**	RUXSOV	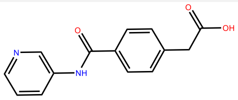	5.154	[77]

**93**	SAHNUO01	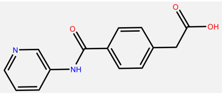	5.123	78
**94**	SIWSOI	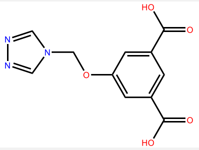	5.266	79
**95**	SOQVOK	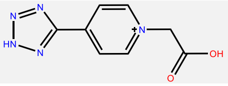	3.444	80
**96**	SOXVIM	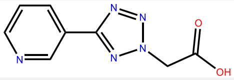	7.889	81
**97**	TAWSER	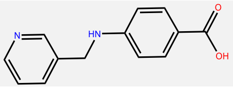	5.543	82
**98**	TAWSIV	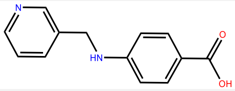	5.105	N/A
**99**	TEDGIT	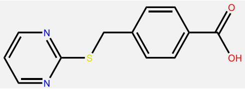	5.528	83
**100**	TOLLIS	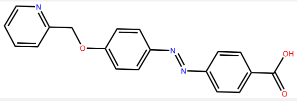	4.767	84
**101**	UGAREA	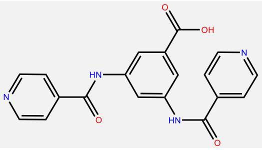	6.668	85
**102**	UGAZOT	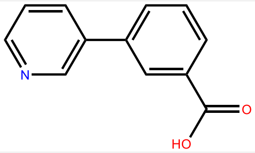	7.607	33
**103**	ULAVUB	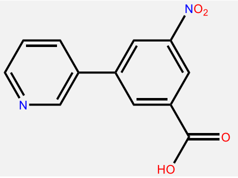	7.787	86
**104**	ULAWAI	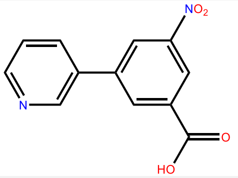	7.722	86
**105**	UMAGAS	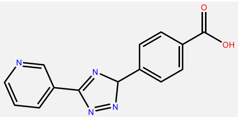	5.627	87
**106**	UNEMUW	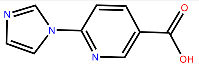	3.995	N/A

**107**	USIBII	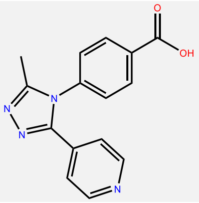	7.915	88
**108**	USIBOO	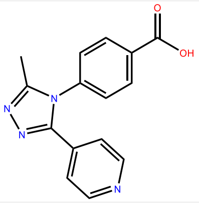	8.401	88
**109**	USIBUU	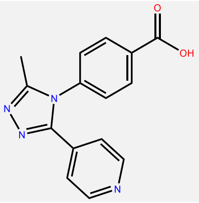	7.535	88
**110**	UZIFEQ	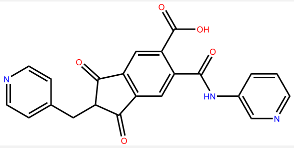	6.796	89
**111**	WAFLIC	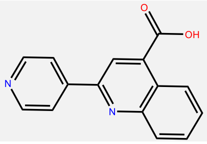	6.357	90
**112**	WAFLOI	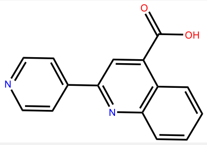	6.515	90
**113**	WAFLUO	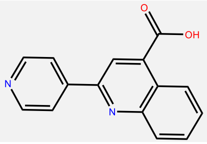	6.604	90
**114**	WOBCOH	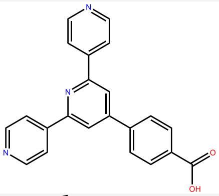	8.825	91
**115**	WUVBOH	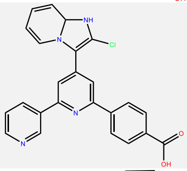	6.557	92
**116**	XIFJIH	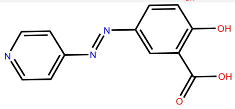	6.380	93

**117**	XIFJON	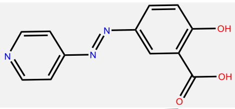	6.455	[93]
**118**	XIFJUT	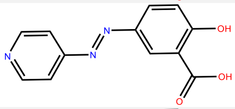	6.600	[93]
**119**	XIFKAA	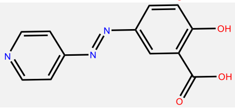	6.381	[93]
**120**	XOBGOM	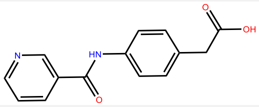	5.676	[94]
**121**	XUKPUR	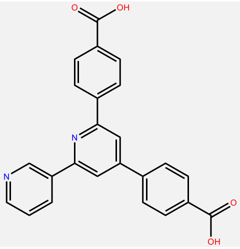	6.627	[95]
**122**	XUKQAY	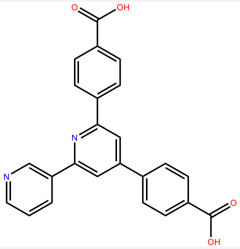	6.323	[95]
**123**	XUKQEC	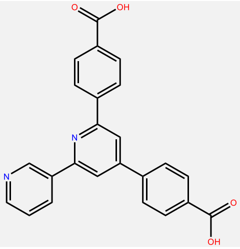	6.525	[95]
**124**	YOQNEA	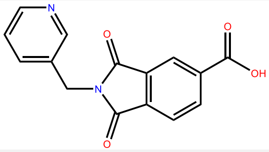	4.878	[96]
**125**	YOQNUQ	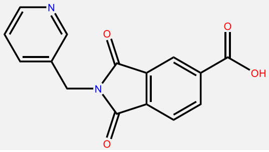	4.770	[96]
**126**	YOVCIX	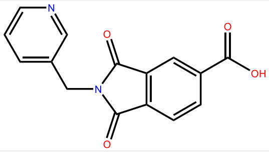	6.970	[97]
**127**	YUNDAN	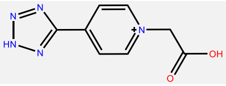	3.447	[98]

**128**	ZINWIF	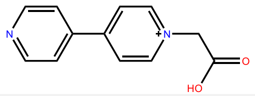	4.262	[99]
**129**	ZINWOL	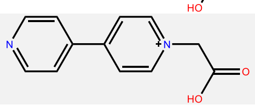	4.453	[99]

4. References

(1) Inc, B. A. *Apex4*; 2012.

(2) Krause, L.; Herbst-Irmer, R.; Sheldrick, G. M.; Stalke, D. Comparison of silver and molybdenum microfocus X-ray sources for single-crystal structure determination. *Journal of Applied Crystallography* **2015**, *48*, 3-10.

(3) Dolomanov, O. V.; Bourhis, L. J.; Gildea, R. J.; Howard, J. A. K.; Puschmann, H. OLEX2: a complete structure solution, refinement and analysis program. *Journal of Applied Crystallography* **2009**, *42* (2), 339-341.

(4) Sheldrick, G. M. SHELXT - Integrated space-group and crystal-structure determination. *Acta Cryst. A* **2015**, *71*, 3-8.

(5) Sheldrick, G. Crystal Structure Refinement with SHELXL. *Acta Crystallographica, Section C: Structural Chemistry* **2015**, *71*, 3-8.

(6) Wang, D.; Wang, T.; Zhao, P.; Shi, Z.; Zhao, Q. Physical characterizations, Hirshfeld surface analysis and luminescent properties of Cd(II) and Pb(II) coordination polymers based on 3-(1,2,4-triazol-1-yl)-benzoic acid. *Inorganica Chimica Acta* **2020**, *508*, 119657. DOI: https://doi.org/10.1016/j.ica.2020.119657.

(7) Zhao, Y.; Zhu, M. Crystal structure of catena-poly[bis(μ2-4-(3-(pyridin-3-yl)-1H-1,2,4-triazol-5-yl)benzoato)-κ2N:O)copper(II)] dihydrate, C28H22N8O6Cu. *Zeitschrift für Kristallographie - New Crystal Structures* **2016**, *231* (1), 179-181. DOI: doi:10.1515/ncrs-2015-0075 (acccessed 2024-11-19).

(8) Hall, G. S.; Angeles, M. J.; Hicks, J.; Turner, D. R. Centric and acentric networks using low-symmetry heterotopic carboxylate/pyridyl ligands. *CrystEngComm* **2016**, *18* (35), 6614-6623, 10.1039/C6CE01000H. DOI: 10.1039/C6CE01000H.

(9) Liao, B.; Li, S.; Yin, X.; Jia, J.; Jiang, Y. Synthesis, Magnetic and Fluorescent Properties of Co(II), Zn(II), and Cd(II) Coordination Polymers Based on N-((3-pyridyl)sulfonyl)aspartic acid. *Chinese Journal of Inorganic Chemistry* **2016**, *32* (7), 1255-1260.

(10) Jiang, Y.; Liu, R.; Gong, Y.; Fan, Y.; Wang, L.; Xu, J. Crystal transformation in Mn(ii) metal–organic frameworks based on a one-dimensional chain precursor. *Dalton Transactions* **2021**, *50* (27), 9540-9546, 10.1039/D1DT00943E. DOI: 10.1039/D1DT00943E.

(11) Lin, W.; Evans, O. R.; Cui, Y. Synthesis and X-ray Structures of Zinc and Cadmium Pyridinecarboxylate Coordination Networks. *Crystal Growth & Design* **2002**, *2* (5), 409-414. DOI: 10.1021/cg0200208.

(12) Ye, X.; Dong, W.; Fang, Y.; Zhao, J.; Li, D. Two NiII/CuII Coordination Polymers Based on Pyridyl-carboxylate Ligand: Synthesis, Crystal Structures, and Magnetic Properties. *Journal of Structural Chemistry* **2018**, *37* (02), 322-328. DOI: 10.14102/j.cnki.0254-5861.2011-1779.

(13) Li, Q.; Wang, H. T.; Ye, Q. One- and two-dimensional CdII coordination polymers constructed from 2-(2-methyl-1H-benzimidazol-1-yl)acetate ligands. *Acta Crystallographica Section C* **2014**, *C70*, 992-997. DOI: 10.1107/S2053229614020853.

(14) Pei, R.-B.; Cao, M.-Y.; Li, L.-K.; Dong, X.-Y.; Zang, S.-Q. A series of transition metal-organic frameworks based on a bipyridinium carboxylate ligand: Syntheses, structures and photoluminescent properties. *Journal of Molecular Structure* **2017**, *1143*, 72-83. DOI: https://doi.org/10.1016/j.molstruc.2017.04.075.

(15) Li, C.-J.; Zheng, S.-R.; Chen, Z.-Y.; Hu, W.-D.; Cai, S.-L.; Fan, J.; Zhang, W.-G. Anion and pH-regulated assembly of three Cd(II) coordination polymers based on 3,5-di(1H-benzo[d]imidazol-1-yl)benzoate. *Journal of Coordination Chemistry* **2017**, *70* (1), 135-144. DOI: 10.1080/00958972.2016.1255882.

(16) Guang-Zhen, L.; Li, Y.-D. Hydrothermal synthesis and crystal structure of catena-poly[diaqua-bis(μ2-4-[(4-pyridinylmethyl)amino]benzoato-κ2N:O)cobalt(II)]–1,2bi(4-pyridyl)ethene–water (1/1/1), C50H50N8O8Co. *Zeitschrift für Kristallographie - New Crystal Structures* **2022**, *237* (4), 719-721. DOI: doi:10.1515/ncrs-2022-0127 (acccessed 2024-11-19).

(17) Wang, D.-W.; Wang, T.; Yan, T.; Du, L.; Zhao, Q.-H. Crystal structure, spectroscopic and thermal properties of copper(II) and manganese(II) coordination polymers based on triazole-benzoic acid ligands. *Transition Metal Chemistry* **2018**, *43* (1), 1-8. DOI: 10.1007/s11243-017-0180-6.

(18) Sheng, Y.-W.; Wang, Y.; Okamura, T.-A.; Sun, W.-Y.; Ueyama, N. Synthesis, crystal structure and nonlinear optical property of cadmium(II) and copper(II) complexes with novel chiral ligand. *Inorganic Chemistry Communications* **2007**, *10* (4), 432-436. DOI: https://doi.org/10.1016/j.inoche.2007.01.001.

(19) Li, S.-L.; Tan, K.; Lan, Y.-Q.; Qin, J.-S.; Li, M.-N.; Du, D.-Y.; Zang, H.-Y.; Su, Z.-M. pH-Dependent Binary Metal−Organic Compounds Assembled from Different Helical Units: Structural Variation and Supramolecular Isomers. *Crystal Growth & Design* **2010**, *10* (4), 1699-1705. DOI: 10.1021/cg9012763.

(20) Ying, S.-M. Syntheses, crystal structures and characterizations of six coordination polymers from reduced Schiff base ligands. *Inorganica Chimica Acta* **2012**, *387*, 366-372. DOI: https://doi.org/10.1016/j.ica.2012.02.029.

(21) Du, M.; Zhao, X.-J.; Wang, Y. Crystal engineering of a versatile building block toward the design of novel inorganic–organic coordination architectures. *Dalton Transactions* **2004**, (14), 2065-2072, 10.1039/B403498H. DOI: 10.1039/B403498H.

(22) Wang, L.-N.; Fu, L.; Zhu, J.-W.; Xu, Y.; Zhang, M.; You, Q.; Wang, P.; Qin, J. Mn(II), Zn(II) and Cd(II) Complexes Based on Oxadiazole Backbone Containing Carboxyl Ligand: Synthesis, Crystal Structure, and Photoluminescent Study. *Acta Chimica Slovenica* **2017**, 202-207. DOI: 10.17344/acsi.2016.3109.

(23) Yan, J.; Zhao, D.; Lu, L. Comparison and Relation between Crystal Structures and Magnetic Properties of Two Manganese(II) Coordination Polymers Based on (Triazol-1-yl)iso/terephthalic Acid. *Chinese Journal of Structural Chemistry* **2021**, *40* (11), 1469-1474. DOI: 10.14102/j.cnki.0254-5861.2011-3186.

(24) Xu, N.; Qiu, X.-H.; Wang, C.; Tang, J.; Cheng, P.; Liao, D.-Z. Construction of Co(II) coordination polymers with flexible carboxylate ligands: structural and magnetic studies. *Inorganic Chemistry Communications* **2013**, *36*, 122-125. DOI: https://doi.org/10.1016/j.inoche.2013.08.021.

(25) Zhong, R.-Q.; Zou, R.-Q.; Du, M.; Jiang, L.; Yamada, T.; Maruta, G.; Takeda, S.; Xu, Q. Metal–organic coordination architectures with 3-pyridin-3-yl-benzoate: crystal structures, fluorescent emission and magnetic properties. *CrystEngComm* **2008**, *10* (5), 605-613, 10.1039/B714491A. DOI: 10.1039/B714491A.

(26) Zou, J.-H.; Zhu, D.-L.; Tian, H.; Li, F. F.; Zhang, F. F.; Yang, G.-W.; Li, Q.-Y.; Miao, Y. X. Construction of six new coordination complexes with 5-(3-pyridyl) tetrazole-2-acetato. *Inorganica Chimica Acta* **2014**, *423*, 87-94. DOI: https://doi.org/10.1016/j.ica.2014.07.034.

(27) Liu, J.; Li, J.; Lu, W. Chromism of three coordination polymers based on 1-(2-carboxyethyl)-4,4′-bipyridinium ligand. *Dyes and Pigments* **2020**, *172*, 107792. DOI: https://doi.org/10.1016/j.dyepig.2019.107792.

(28) Hawes, C. S.; Moubaraki, B.; Murray, K. S.; Kruger, P. E.; Turner, D. R.; Batten, S. R. Exploiting the Pyrazole-Carboxylate Mixed Ligand System in the Crystal Engineering of Coordination Polymers. *Crystal Growth & Design* **2014**, *14* (11), 5749-5760. DOI: 10.1021/cg501004u.

(29) Xi, Y.; Wei, W.; Xu, Y.; Huang, X.; Zhang, F.; Hu, C. Coordination Polymers Based on Substituted Terpyridine Ligands: Synthesis, Structural Diversity, and Highly Efficient and Selective Catalytic Oxidation of Benzylic C–H Bonds. *Crystal Growth & Design* **2015**, *15* (6), 2695-2702. DOI: 10.1021/acs.cgd.5b00008.

(30) Su, Z.; Wang, Z.-B.; Sun, W.-Y. Syntheses, structures, and properties of lead(II) and nickel(II) complexes with 3,5-di(1H-imidazol-1-yl)benzoate. *Journal of Coordination Chemistry* **2011**, *64* (1), 170-178. DOI: 10.1080/00958972.2010.537332.

(31) Huang, T.; Wang, Y.-L.; Yin, Q.; Karadeniz, B.; Li, H.-F.; Lü, J.; Cao, R. Cobalt coordination polymers regulated by in situ ligand transformation. *CrystEngComm* **2016**, *18* (15), 2742-2747, 10.1039/C6CE00104A. DOI: 10.1039/C6CE00104A.

(32) Tang, L.; Wu, Y.-P.; Fu, F.; Hou, X.-Y.; Wei, Q.-B. <i>catena</i>-Poly[zinc(II)-bis[μ₂-3-(3-pyridyl)benzoato]-κ²<i>O</i>:<i>N</i>;κ²<i>N</i>:<i>O</i>]. *Acta Crystallographica Section E Structure Reports Online* **2011**, *67* (7), m894-m894. DOI: 10.1107/s1600536811021404.

(33) Du, J.; Zou, G.; Wang, X.; Xie, B. Solvent-Induced Bond Rearrangement in Two Zn(II) Coordination Polymers and The Single-Crystal- to-Single- Crystal Transformation. *Synthesis and Reactivity in Inorganic, Metal-Organic, and Nano-Metal Chemistry* **2014**, *44* (6), 793-797. DOI: 10.1080/15533174.2013.790439.

(34) Hong, D.-F.; Li, M.-F.; Chu, T.-T. Crystal structure of catena-poly[aqua-bis[μ2-6-(1H-imidazol-1-yl)nicotinato-κ2 N,O]copper(II)], C18H14N6O5Cu. *Zeitschrift für Kristallographie - New Crystal Structures* **2022**, *237* (3), 453-454. DOI: doi:10.1515/ncrs-2022-0059 (acccessed 2024-11-19).

(35) Du, M.; Li, C.-P. Metal-directed 1-D molecular-box based coordination polymers with mono- and di-nuclear nodes – Construction of 3-D supramolecular networks via hydrogen bonding and S···S interactions. *Inorganica Chimica Acta* **2006**, *359* (5), 1690-1696. DOI: https://doi.org/10.1016/j.ica.2005.11.038.

(36) Zhang, Y.; Wang, J.; Zheng, L.; Hu, S.; Lin, Z.; Tong, M. Structural diversity and reactivity of d10 metal-(4-pyridylthio)acetate system. *Chinese Science Bulletin* **2009**, *54* (23), 4277-4284. DOI: 10.1007/s11434-009-0673-6.

(37) Lässig, D.; Lincke, J.; Gerhardt, R.; Krautscheid, H. Solid-State Syntheses of Coordination Polymers by Thermal Conversion of Molecular Building Blocks and Polymeric Precursors. *Inorganic Chemistry* **2012**, *51* (11), 6180-6189. DOI: 10.1021/ic300235s.

(38) Dzesse T, C. N.; Nfor, E. N.; Bourne, S. A. Vapor Sorption and Solvatochromism in a Metal–Organic Framework of an Asymmetric Pyridylcarboxylate. *Crystal Growth & Design* **2018**, *18* (1), 416-423. DOI: 10.1021/acs.cgd.7b01417.

(39) Xin, W.; Qingyun, L.; Liguo, Y.; Nana, L.; Yuqiang, D. Crystal structure of catena-poly[diaqua-bis(μ2-1H-benzo[d]imidazole-5-carboxylato-κ2N:O)nickel(II)] dihydrate, C16H18N4NiO8. *Zeitschrift für Kristallographie - New Crystal Structures* **2018**, *233* (2), 203-204. DOI: doi:10.1515/ncrs-2017-0199 (acccessed 2024-11-19).

(40) Luo, X.-P.; Han, L. catena-Poly[[diaquanickel(II)]-bis[[mu]-(4-pyridylsulfanyl)acetato-[kappa]2N:O;[kappa]2O:N]]. *Acta Crystallographica Section E* **2007**, *63* (6), m1745. DOI: 10.1107/S1600536807023550.

(41) Sun, J.-K.; Tan, B.; Cai, L.-X.; Chen, R.-P.; Zhang, J.; Zhang, J. Polycatenation-Driven Self-Assembly of Nanoporous Frameworks Based on a 1D Ribbon of Rings: Regular Structural Evolution, Interpenetration Transformation, and Photochemical Modification. *Chemistry – A European Journal* **2014**, *20* (9), 2488-2495. DOI: https://doi.org/10.1002/chem.201303700.

(42) Tai, X.-S.; Wang, X.; Li, P.-F. Synthesis, Crystal Structure, and Luminescent Property of a Cd(II) Coordination Polymer with a N-Nicotinoylglycine Ligand. *Crystals* **2017**, *7* (2), 33.

(43) Bettle, P. J.; Dawe, L. N.; Anwar, M. U.; Thompson, L. K. Dinuclear, Tetranuclear and Chain (MnII, CoII) Complexes of Multifunctional Hydrazone Ligands – Structural and Magnetic Studies. *European Journal of Inorganic Chemistry* **2011**, *2011* (32), 5036-5042. DOI: https://doi.org/10.1002/ejic.201100746.

(44) Cheng, Y.; Yang, M.-L.; Hu, H.-M.; Xu, B.; Wang, X.; Xue, G. Syntheses, structures and luminescence for zinc coordination polymers based on a multifunctional 4′-(3-carboxyphenyl)-3,2′:6′,3″-terpyridine ligand. *Journal of Solid State Chemistry* **2016**, *239*, 121-130. DOI: https://doi.org/10.1016/j.jssc.2016.04.002.

(45) Wu, B.-L.; Wang, R.-Y.; Zhang, H.-Y.; Hou, H.-W. Coordination polymers of unsymmetrical angular ligand 3-pyridin-4-ylbenzoate acid: Syntheses, structural diversity and properties. *Inorganica Chimica Acta* **2011**, *375* (1), 2-10. DOI: https://doi.org/10.1016/j.ica.2011.05.029.

(46) Zhang, R.; Zhu, W.; Zhang, M. Hydrothermal Synthesis and Crystal Structure of Ni (II) Complex with 4-(4-Pyridyl) isophthalic Acid. *Asian Journal of Chemistry* **2015**, *27* (6), 2347.

(47) Yang, W.-C.; Zheng, Z.-B.; Sun, H.-L.; Wang, K.-Z. Synthesis, crystal structure, and properties of a double-helical zinc(II) coordination polymer with Ozagrel drug. *Spectrochimica Acta Part A: Molecular and Biomolecular Spectroscopy* **2012**, *86*, 187-190. DOI: https://doi.org/10.1016/j.saa.2011.10.022.

(48) Yang, J.; Shen, L.; Yang, G.-W.; Li, Q.-Y.; Shen, W.; Jin, J.-N.; Zhao, J.-J.; Dai, J. A set of new transition metal-based coordination complexes dependent upon Hpztza ligand (Hpztza=2-(5-(pyrazin-2-yl)-2H-tetrazol-2-yl) acetic acid). *Journal of Solid State Chemistry* **2012**, *186*, 124-133. DOI: https://doi.org/10.1016/j.jssc.2011.11.017.

(49) Han, L.; Gong, Y.; Yuan, D.; Hong, M. Luminescent 2D supramolecular network constructed from tubular coordination polymer based on H-bonding and π–π interactions. *Journal of Molecular Structure* **2006**, *789* (1), 128-132. DOI: https://doi.org/10.1016/j.molstruc.2005.12.032.

(50) Zhang, Z.-H.; Du, M. Flexible and versatile anionic modules in the direction of 1-D, 2-D, and 3-D coordination frameworks by metal–ligand synergistic interactions. *CrystEngComm* **2008**, *10* (10), 1350-1357, 10.1039/B803736A. DOI: 10.1039/B803736A.

(51) Wang, X.-J.; Huang, T.-H.; Tang, L.-H.; Cen, Z.-M.; Ni, Q.-L.; Gui, L.-C.; Jiang, X.-F.; Liu, H.-K. Synthesis and structures of helical and meso-helical coordination polymers directed by the conformation restriction of flexible/angular pyridine-containing ligands. *CrystEngComm* **2010**, *12* (12), 4356-4364, 10.1039/C0CE00110D. DOI: 10.1039/C0CE00110D.

(52) Yue, D.; Li, G.-Y.; Ma, X.-R.; Fu, W.-P.; Zou, Y.-P.; Qin, L.; Zhang, M.-D. Two Co/Ni(II)-Based Coordination Polymers for Degradation of Dyes. *Crystallography Reports* **2021**, *66* (7), 1262-1267. DOI: 10.1134/S1063774521070233.

(53) Zhao, H.-Q.; Yang, S.-P.; Ding, N.-N.; Qin, L.; Qiu, G.-H.; Chen, J.-X.; Zhang, W.-H.; Chen, W.-H.; Hor, T. S. A. A zwitterionic 1D/2D polymer co-crystal and its polymorphic sub-components: a highly selective sensing platform for HIV ds-DNA sequences. *Dalton Transactions* **2016**, *45* (12), 5092-5100, 10.1039/C5DT04410C. DOI: 10.1039/C5DT04410C.

(54) Liu, M.-L.; Ren, P.; Shi, W.; Cui, J. Z.; Cheng, P.; Gao, H.-L. Synthesis, structures and magnetic properties of 1D to 3D coordinated polymers based on series of flexible sulfide ligands. *Inorganica Chimica Acta* **2011**, *378* (1), 56-65. DOI: https://doi.org/10.1016/j.ica.2011.08.022.

(55) Wang, J.-G. Crystal structure of catena-poly{diaqua-bis(3-(1H-1,2,4-triazol-1-yl)benzoato-κ2O:N)copper(II)} monohydrate, C18H18CuN6O7. *Zeitschrift für Kristallographie - New Crystal Structures* **2017**, *232* (4), 579-580. DOI: doi:10.1515/ncrs-2016-0350 (acccessed 2024-11-19).

(56) Liu, P.; Zhu, Y.; Xia, L.; Wu, Y.-L.; Lu, X.-Q.; Xia, C.-K.; Xie, J.-M. Syntheses, structures, fluorescence and heterogeneous catalyses of three coordination polymers based on 4-benzoimidazol-1-yl-methyl benzoic acid. *Polyhedron* **2015**, *102*, 276-283. DOI: https://doi.org/10.1016/j.poly.2015.07.004.

(57) Xu, G.-J.; Zhao, Y.-H.; Shao, K.-Z.; Lan, Y.-Q.; Wang, X.-L.; Su, Z.-M.; Yan, L.-K. Secondary ligand-directed assembly of ZnII and CdII coordination architectures: From 1D to 3D compounds based on pyridine carboxylate ligands. *Journal of Molecular Structure* **2010**, *983* (1), 93-98. DOI: https://doi.org/10.1016/j.molstruc.2010.08.037.

(58) Wang, T.-T.; Zhang, J.-L.; Hu, H.-M.; Cheng, Y.; Xue, L.-L.; Wang, X.; Wang, B.-Z. Syntheses, structures and luminescent properties of Zn/Cd coordination polymers based on 4′-(2-carboxyphenyl)-3,2′:6′,3″-terpyridine. *Polyhedron* **2018**, *151*, 43-50. DOI: https://doi.org/10.1016/j.poly.2018.05.017.

(59) Guo, F. Synthesis and crystal structures of pH-dependent Mn(II) coordination polymers with 3-pyrid-3-ylbenzoic acid. *Journal of Coordination Chemistry* **2009**, *62* (22), 3606-3612. DOI: 10.1080/00958970903154496.

(60) Li, K.-H.; Liu, C. The crystal structure of catena-poly[(μ2-4-(benzo[d]imidazol-2-yl)benzenecarboxylato-κ2N,O)-(μ2-4-(benzo[d]imidazol-2-yl)benzenecarboxylato-κ3N,O:O′)cadmium(II)]dihydrate, C28H22CdN4O6. *Zeitschrift für Kristallographie - New Crystal Structures* **2020**, *235* (5), 1033-1035. DOI: doi:10.1515/ncrs-2020-0149 (acccessed 2024-11-19).

(61) Wang, Y.; Wen, R.-M.; Su, Y.-H.; Wang, J.-R.; Gong, S.-M.; Zhou, R.-S.; Yang, Q.-F.; Song, J.-F. A new zinc-based coordination polymer with blue light emission: synthesis, crystal structure and multifunctional fluorescence sensing properties. *Journal of Molecular Structure* **2022**, *1264*, 133154. DOI: https://doi.org/10.1016/j.molstruc.2022.133154.

(62) Fan, J.; Zhang, Y.-A.; Okamura, T.-a.; Zou, Z.-H.; Ueyama, N.; Sun, W.-Y. Synthesis and crystal structure of a one-dimensional coordination polymer of nickel(II) with 4′-(imidazol-1-ylmethyl)benzoate anion. *Inorganic Chemistry Communications* **2001**, *4* (9), 501-503. DOI: https://doi.org/10.1016/S1387-7003(01)00259-3.

(63) Zheng, L.-N.; Cheng, Y.; Hu, H.-M.; Bai, C.; Wang, X.; Xue, G. Syntheses, structures and magnetic properties for transition metal coordination polymers based on polycarboxylate and isomeric terpyridyl carboxylate ligands. *Journal of Solid State Chemistry* **2019**, *272*, 210-220. DOI: https://doi.org/10.1016/j.jssc.2019.02.004.

(64) Paul, M.; Dastidar, P. Coordination polymers derived from pyridyl carboxylate ligands having an amide backbone: an attempt towards the selective separation of CuII cation following

in situ crystallization under competitive conditions. *CrystEngComm* **2014**, *16* (33), 7815-7829, 10.1039/C4CE00733F. DOI: 10.1039/C4CE00733F.

(65) Duan, L.; Wu, X.-W.; Ma, J.-P. Poly[[{μ₂-3-[(1<i>H</i>-benzimidazol-1-yl)methyl]benzoato}cadmium(II)] 0.1-hydrate]. *Acta Crystallographica Section E Structure Reports Online* **2010**, *66* (5), m530-m530. DOI: 10.1107/s1600536810013292.

(66) Zhao, Y.; Guo, D.-J. Crystal structure of catena-poly[diaqua-bis-(μ2-5-carboxy-2-(pyridin-4-yl)benzoato-κ2O:N)cadmium(II)] dihydrate, C26H24N2O12Cd. *Zeitschrift für Kristallographie - New Crystal Structures* **2016**, *231* (1), 41-42. DOI: doi:10.1515/ncrs-2014-9092 (acccessed 2024-11-19).

(67) Wang, Y.; Tian, Y.; Luo, J. Two new d10 metal-directed coordination polymers based on an unsymmetrical ligand 3-pyrimidin-5-ylbenzoic acid. *Inorganic Chemistry Communications* **2011**, *14* (8), 1258-1261. DOI: https://doi.org/10.1016/j.inoche.2011.04.036.

(68) Kuai, H.-W.; Cheng, X.-C.; Zhu, X.-H. Synthesis, characterization, and crystal structures of two coordination polymers from 3,5-bis(pyridin-4-ylmethyl) aminobenzoic acid. *Journal of Coordination Chemistry 64* (19), 3323-3332. DOI: 10.1080/00958972.2011.608846.

(69) Gao, R.-Q.; Xia, C.-H.; Li, G.-T. catena-Poly[[diaquanickel(II)]-bis(μ-2-{[5-(pyridin-4-yl)-1,3,4-oxadiazol-2-yl]sulfanyl}acetato)]. *Acta Crystallographica Section E Structure Reports Online* **2012**, *68* (6), m763-m764. DOI: 10.1107/s1600536812020259.

(70) Cao, K.-L.; Zhang, Y.-P.; Cai, Y.-N.; Xu, X.-W.; Feng, Y.-L. Secondary ligand-directed assembly of Co(II) coordination polymers based on a pyridine carboxylate ligand. *Journal of Solid State Chemistry* **2014**, *215*, 34-42. DOI: https://doi.org/10.1016/j.jssc.2014.03.025.

(71) Xiong, P.; Li, J.; Bu, H.; Wei, Q.; Zhang, R.; Chen, S. Copper(II) complexes with 4-(1H-1, 2, 4-trizol-1-ylmethyl) benzoic acid: Syntheses, crystal structures and antifungal activities.

Journal of Solid State Chemistry **2014**, *215*, 292-299. DOI: https://doi.org/10.1016/j.jssc.2014.04.012.

(72) Chen, S.-S.; Liu, Q.; Zhao, Y.; Qiao, R.; Sheng, L.-Q.; Liu, Z.-D.; Yang, S.; Song, C.-F. New Metal–Organic Frameworks Constructed from the 4-Imidazole-Carboxylate Ligand: Structural Diversities, Luminescence, and Gas Adsorption Properties. *Crystal Growth & Design* **2014**, *14* (8), 3727-3741. DOI: 10.1021/cg401811c.

(73) Twaróg, K.; Hołyńska, M.; Kochel, A. A new photoluminescent coordination polymer constructed with an N-donor ligand having extended coordination capabilities derived from quinoline and pyridine. *Acta Crystallographica Section C* **2020**, *76* (5), 500-506. DOI: https://doi.org/10.1107/S2053229620004593.

(74) Wang, D.; Wang, T.; Yan, T.; Du, L.; Zhao, Q. Structural and spectroscopic characterization of two cadmium (II) complexes based on [1,2,4]-triazole derivatives. *Chinese Journal of Inorganic Chemistry* **2017**, *33* (8), 1443-1449.

(75) Liu, J.-j.; Li, J. Photochromism of four 1D coordination polymers based on 1-(2-carboxyethyl)-4,4′-bipyridinium ligand. *Dyes and Pigments* **2019**, *170*, 107552. DOI: https://doi.org/10.1016/j.dyepig.2019.107552.

(76) Deng, M.; Mukherjee, S.; Liang, Y.-J.; Fang, X.-D.; Zhu, A.-X.; Zaworotko, M. J. Water vapour induced reversible switching between a 1-D coordination polymer and a 0-D aqua complex. *Chemical Communications* **2022**, *58* (59), 8218-8221, 10.1039/D2CC02777A. DOI: 10.1039/D2CC02777A.

(77) Paul, A.; Das, K.; Karmakar, A.; Guedes da Silva, M. F. C.; Pombeiro, A. J. L. A mechanistic insight into the rapid and selective removal of Congo Red by an amide

functionalised Zn(ii) coordination polymer. *Dalton Transactions* **2020**, *49* (37), 12970-12984, 10.1039/D0DT02172E. DOI: 10.1039/D0DT02172E.

(78) Paul, A.; Upadhyay, K. K.; Backović, G.; Karmakar, A.; Vieira Ferreira, L. F.; Šljukić, B.; Montemor, M. F.; Guedes da Silva, M. F. C.; Pombeiro, A. J. L. Versatility of Amide-Functionalized Co(II) and Ni(II) Coordination Polymers: From Thermochromic-Triggered Structural Transformations to Supercapacitors and Electrocatalysts for Water Splitting. *Inorganic Chemistry* **2020**, *59* (22), 16301-16318. DOI: 10.1021/acs.inorgchem.0c02084.

(79) Yang, Y.; Yang, J.; Du, P.; Liu, Y.-Y.; Ma, J.-F. A series of coordination polymers constructed by the semi-rigid bifunctional ligand 5-((1H-1,2,4-triazol-1-yl)methoxy) isophthalic acid: syntheses, structures and the role of solvents. *CrystEngComm* **2014**, *16* (6), 1136-1148, 10.1039/C3CE42048E. DOI: 10.1039/C3CE42048E.

(80) Yang, G.-W.; Li, Q.-Y.; Zhou, Y.; Gu, G.-Q.; Ma, Y.-S.; Yuan, R.-X. Three Mn(II) supramolecular coordination complexes containing carboxylate–tetrazolate ligands. *Inorganica Chimica Acta* **2009**, *362* (4), 1234-1238. DOI: https://doi.org/10.1016/j.ica.2008.06.011.

(81) Zou, J. H.; Tian, H.; Wang, Z.; Li, F. S.; Zhang, F. F.; Zhao, L. Y.; Zhang, P.; Zhu, D. L.; Yang, G. W.; Li, Q. Y. Coordination Architectures of Manganese Complexes based on the Liagnd 3-Pytza or Mixed Ligands [3-Pytza = 5-(3-Pyridyl)tetrazole-2-acetato]. *Zeitschrift für anorganische und allgemeine Chemie* **2014**, *640* (12-13), 2566-2570. DOI: https://doi.org/10.1002/zaac.201400252.

(82) Liu, B.; Fan, L.-J.; Liu, Y.-Y.; Yang, J.; Ma, J.-F. Syntheses and structures of Cd(II) and Co(II) compounds of 4-[(3-pyridyl)methylamino]benzoate anion. *Journal of Coordination Chemistry* **2011**, *64* (3), 413-423. DOI: 10.1080/00958972.2010.548520.

(83) Han, L.; Yuan, D.; Wu, B.; Liu, C.; Hong, M. Syntheses, structures and properties of three novel coordination polymers with a flexible asymmetrical bridging ligand. *Inorganica Chimica Acta* **2006**, *359* (7), 2232-2240. DOI: https://doi.org/10.1016/j.ica.2005.12.077.

(84) Zhao, Y. Crystal structure of catena-poly[diaqua-bis(μ2-4-((4-(pyridin-2-ylmethoxy)phenyl)diazenyl)benzoato-κ3O,O′:N)cadmium(III)], Cd(C19H14O3N3)2(H2O). *Zeitschrift für Kristallographie - New Crystal Structures* **2019**, *234* (4), 741-742. DOI: doi:10.1515/ncrs-2019-0083 (acccessed 2024-11-19).

(85) Chen, M.-S.; Chen, S.-S.; Okamura, T.-A.; Su, Z.; Sun, W.-Y.; Ueyama, N. Syntheses and crystal structures of two supramolecular isomers of manganese(II) with 3,5-bis(isonicotinamido)benzoate. *Journal of Coordination Chemistry* **2009**, *62* (15), 2421-2428. DOI: 10.1080/00958970902870902.

(86) Tang, L.; Wang, H.; Fu, Y.; Hou, X.; Wang, X.; Wang, J. Two luminescent d10 metal coordination polymers based on 3-nitro-5-(pyridin-3-yl)benzoic acid. *Zeitschrift für Naturforschung B* **2021**, *76* (1), 65-69. DOI: doi:10.1515/znb-2020-0163 (acccessed 2024-11-19).

(87) Du, P.-Y.; Su, J.; Lv, R.; Gu, W.; Liu, X. A series of novel complexes derived from 4-(5-(pyridin-3-yl)-4H-1,2,4-triazol-3-yl) benzoic acid: Hydrothermal syntheses, crystal structures, and properties. *Polyhedron* **2016**, *115*, 86-91. DOI: https://doi.org/10.1016/j.poly.2016.04.018.

(88) Lässig, D.; Lincke, J.; Griebel, J.; Kirmse, R.; Krautscheid, H. Synthesis, Crystal Structure, and Electron Paramagnetic Resonance Investigations of Heteronuclear CoII/ZnII and CoII/CdII Coordination Polymers. *Inorganic Chemistry* **2011**, *50* (1), 213-219. DOI: 10.1021/ic101783z.

(89) Li, G.-B.; Cheng, J.-Q.; Pan, R.-K.; Liu, S.-G. Manganese(II) and cobalt(II) complexes based on pyridyl diimide: in situ ligand formation, crystal structures, and sorption properties. *Transition Metal Chemistry* **2016**, *41* (8), 917-922. DOI: 10.1007/s11243-016-0095-7.

(90) Zhang, L.; Man, Z.-W.; Zhang, Y.; Hong, J.; Guo, M.-R.; Qin, J. Synthesis, Structure Evaluation, Spectroscopic and Antibacterial Investigation of Metal Complexes with 2-(Pyridin-4-yl)quinoline-4-carboxylic Acid. *Acta Chimica Slovenica* **2016**, 891-898. DOI: 10.17344/acsi.2016.2895.

(91) Wang, M.-S.; Li, M.-X.; He, X.; Shao, M.; Wang, Z.-X. Synthesis, crystal structure and magnetic properties of two coordination polymers with 4-(4-carboxyphenyl)-4,2′:6′,4″-terpyridine ligand. *Inorganic Chemistry Communications* **2014**, *42*, 38-41. DOI: https://doi.org/10.1016/j.inoche.2014.01.017.

(92) Li, B.; Yan, Q.-Q.; Yong, G.-P. A new porous coordination polymer reveals selective sensing of Fe3+, Cr2O72−, CrO42−, MnO4− and nitrobenzene, and stimuli-responsive luminescence color conversions. *Journal of Materials Chemistry C* **2020**, *8* (34), 11786-11795, 10.1039/C9TC07030C. DOI: 10.1039/C9TC07030C.

(93) Mishra, R.; Ahmad, M.; Tripathi, M. R. Four novel isostructural coordination polymers {[M(H′L)2(H2O)2]·2DMF} [M=Zn(II), Cd(II), Mn(II) and Co(II)] built using a nitrogen and oxygen donor azo ligand: Crystal structures and fluorescence studies. *Polyhedron* **2013**, *54*, 189-195. DOI: https://doi.org/10.1016/j.poly.2013.02.044.

(94) Zheng, Z. N.; Lee, S. W. A pyridyl–carboxylate type linking ligand containing an intervening amide group and its cadmium and nickel coordination polymers: (3-py)–C(O)NH–C6H4–CH2–COOH (HL), {[CdL2](H2O)}∞, and {[NiL2(H2O)2](H2O)}∞. *Polyhedron* **2014**, *69*, 197-204. DOI: https://doi.org/10.1016/j.poly.2013.12.002.

(95) Li, B.; Yan, Q.-Q.; Yong, G.-P. Crystal structures and properties of four coordination polymers based on a new asymmetric ligand: Tuning structure/dimensionality by various organic solvents. *Inorganica Chimica Acta* **2020**, *503*, 119403. DOI: https://doi.org/10.1016/j.ica.2019.119403.

(96) Wu, J.-Y.; Yuan, P.-T.; Hsiao, C.-C.; Chang, H.-K.; Liu, Y.-C.; Hsu, L.-J.; Chiang, M.-H. Structural diversity in polymeric and discrete complexes constructed by divalent transition metals and unsymmetrical quasi semirigid pyridinecarboxylate isomers. *Journal of Solid State Chemistry* **2019**, *277*, 701-712. DOI: https://doi.org/10.1016/j.jssc.2019.07.033.

(97) Wu, J.-Y.; Hsiao, C.-C.; Chiang, M.-H. Concomitant Crystallization of Genuine Supramolecular Isomeric Rhombus Grid and Ribbon. *Crystal Growth & Design* **2014**, *14* (9), 4321-4328. DOI: 10.1021/cg500380v.

(98) Yang, G.-W.; Ma, Y.-S.; Li, Q.-Y.; Zhou, Y.; Gu, G.-Q.; Wu, Y.; Yuan, R.-X. U(VI) and Zn(II) coordination complexes with 5-[N-acetato(4-pyridyl)]tetrazolate anions. *Journal of Coordination Chemistry* **2009**, *62* (11), 1766-1774. DOI: 10.1080/00958970802705893.

(99) Liu, J. Three 1D coordination polymers based on bipyridinium carboxylate ligands: Photochromism. *Dyes and Pigments* **2019**, *160*, 476-482. DOI: https://doi.org/10.1016/j.dyepig.2018.08.042.
